# Roles of Localized Electronic Structures Caused by π Degeneracy Due to Highly Symmetric Heavy Atom‐Free Conjugated Molecular Crystals Leading to Efficient Persistent Room‐Temperature Phosphorescence

**DOI:** 10.1002/advs.201900410

**Published:** 2019-05-10

**Authors:** Shuzo Hirata

**Affiliations:** ^1^ Department of Engineering Science University of Electro‐Communications 1‐5‐1 Chofugaoka Chofu Tokyo 182‐8585 Japan

**Keywords:** aggregation induced emission, persistent room‐temperature phosphorescence, spin–orbit coupling, transfer integral, triplet exciton diffusion

## Abstract

Conjugated molecular crystals with persistent room‐temperature phosphorescence (RTP) are promising materials for sensing, security, and bioimaging applications. However, the electronic structures that lead to efficient persistent RTP are still unclear. Here, the electronic structures of tetraphenylmethane (C(C_6_H_5_)_4_), tetraphenylsilane (Si(C_6_H_5_)_4_), and tetraphenylgermane (Ge(C_6_H_5_)_4_) showing blue‐green persistent RTP under ambient conditions are investigated. The persistent RTP of the crystals originates from minimization of triplet exciton quenching at room temperature not suppression of molecular vibrations. Localization of the highest occupied molecular orbitals (HOMOs) of the steric and highly symmetric conjugated crystal structures decreases the overlap of intermolecular HOMOs, minimizing triplet exciton migration, which accelerates defect quenching of triplet excitons. The localization of the HOMOs over the highly symmetric conjugated structures also induces moderate charge‐transfer characteristics between high‐order singlet excited states (S*_m_*) and the ground state (S_0_). The combination of the moderate charge‐transfer characteristics of the S*_m_*–S_0_ transition and local‐excited state characteristics between the lowest excited triplet state and S_0_ accelerates the phosphorescence rate independent of the vibration‐based nonradiative decay rate from the triplet state at room temperature. Thus, the decrease of triplet quenching and increase of phosphorescence rate caused by the HOMO localization contribute to the efficient persistent RTP of Ge(C_6_H_5_)_4_ crystals.

## 1 Introduction

Room‐temperature phosphorescence (RTP) is used in organic light‐emitting diodes,^1^, ^2^, ^3^, ^4^ photodynamic therapy,^5^, ^6^ in vivo imaging,^7^ and sensing applications.^8^ The highly efficient RTP from heavy‐atom complexes is caused by a fast radiative process from the lowest triplet excited state (T_1_).^1^, ^2^, ^3^, ^4^, ^5^, ^6^, ^7^, ^8^ In contrast, the radiative rate from T_1_ (*k*_p_) of heavy atom‐free conjugated structures is small. In addition, the nonradiative rate of intramolecular vibrational relaxation at room temperature (RT) from T_1_ (*k*_nr_(RT)) and the triplet quenching rate at RT caused by interactions with the ambient surroundings (*k*_q_(RT)) are often much larger than *k*_p_. Therefore, reports of RTP from heavy metal‐free aromatic molecules under ambient conditions have been scarce.^9^, ^10^ In the last five years, the fast nonradiative processes from T_1_ have been suppressed in a variety of heavy atom‐free conjugated molecules under ambient conditions, which has allowed electrons in T_1_ to partly access the slow RTP pathway.^11^, ^12^, ^13^, ^14^, ^15^, ^16^, ^17^ Because heavy atom‐free molecules with T_1_ with strong ππ* characteristics have very small *k*_p_,^18^ RTP from such conjugated structures exhibits persistent emission characteristics.^12^, ^13^, ^14^, ^15^, ^16^, ^17^ Persistent emission characteristics from such materials could be used in small‐scale and cost‐effective 2D photodetectors.^19^ Therefore, these materials are potentially useful for a variety of applications, such as thermometers,^20^ security,^13^, ^15^, ^21^, ^22^, ^23^, ^24^ stimuli sensors,^25^, ^26^ optical recording,^27^, ^28^, ^29^ and bioimaging,^28^, ^30^, ^31^ which are independent of autofluorescence. In 1939, Clapp reported that tetraphenylmethane (C(C_6_H_5_)_4_), tetraphenylsilane (Si(C_6_H_5_)_4_), and tetraphenylgermane (Ge(C_6_H_5_)_4_) as nonpolar highly symmetric aromatic molecular crystals showed persistent RTP under ambient conditions.^9^ Since then, persistent RTP has been observed under vacuum or inert conditions but disappeared under ambient conditions for some other aromatic structures.^32^, ^33^, ^34^, ^35^, ^36^, ^37^, ^38^ Except for a few reports before 2000,^9^, ^10^ persistent RTP characteristics under ambient conditions have been observed recently from heavy atom‐free isolated conjugated molecules doped in a highly rigid amorphous host^12^, ^20^, ^21^, ^39^, ^40^, ^41^ and crystalline host,^42^, ^43^ carbon nanodots,^13^, ^22^, ^23^, ^24^, ^44^, ^45^ heavy atom‐free aromatic crystals,^14^, ^15^, ^16^, ^25^, ^26^, ^46^, ^47^, ^48^, ^49^, ^50^, ^51^, ^52^ metal–organic frameworks,^17^, ^53^ and nonconventional luminogens.^54^, ^55^

Considering host–guest materials, in 2013 we reported efficient red–green–blue persistent RTP with more than 10% efficiency from heavy atom‐free conjugated structures doped into highly rigid short conjugated host molecules under ambient conditions.^12^ In the host–guest molecular materials, the highly rigid short conjugated matrix could suppress *k*_q_(RT) caused by endothermic triplet–triplet energy transfer from guest to host molecules and effectively protect triplet excited species from oxygen, contributing substantially to the appearance of persistent RTP. Quantum chemical calculations of such rigid hosts also revealed that *k*_nr_(RT) based on free intramolecular vibrations is intrinsically small and approaches a very small *k*_p_.^56^ However, for most heavy atom‐free conjugated molecular crystals with persistent RTP,^9^, ^14^, ^15^, ^16^, ^25^, ^26^, ^46^, ^47^, ^48^, ^49^, ^50^, ^51^ the quantum yield of persistent RTP (*Φ*_p_(RT)) of conjugated molecular crystals with an RTP lifetime approaching to 1 s is often a few percent or less.^57^, ^58^ Small *k*_nr_(RT) caused by the suppression of intramolecular vibrations of target chromophores by the strong intermolecular interactions in the crystalline packing has often been considered as a candidate for the origin of the appearance of persistent RTP. However, very recently, cooperative analysis using microscopy and quantum chemical calculations indicated that suppressed triplet diffusion caused by the weak interaction of the molecular orbitals (MOs) related to the transition from T_1_ to the ground state (S_0_) greatly decreases *k*_q_(RT) is the main cause of the appearance of persistent RTP.^59^ This indicates that the large decrease of *k*_q_(RT) contributes to the weak persistent RTP while the small *Φ*_p_(RT) of most aromatic crystals with persistent RTP characteristics is intrinsically because *k*_p_ < *k*_nr_(RT). Therefore, a way to increase *k*_p_ without increasing *k*_nr_(RT) is crucial to obtain much larger *Φ*_p_(RT). However, an overall discussion of *k*_p_, *k*_nr_(RT), and *k*_q_(RT) and an approach to increase *k*_p_ independent of *k*_nr_(RT) in conjugated molecular crystals have not been reported as yet.

Here we investigate the electronic structures controlling *k*_p_, *k*_nr_(RT), and *k*_q_(RT) of nonpolar and highly symmetric conjugated molecular crystals showing persistent blue‐green RTP in air. C(C_6_H_5_)_4_, Si(C_6_H_5_)_4_, and Ge(C_6_H_5_)_4_ as the steric and highly symmetric aromatic structures do not show RTP in degassed solution, whereas they show persistent RTP under ambient conditions in the crystalline state.^9^ The RTP lifetimes (τ_p_(RT)) of C(C_6_H_5_)_4_, Si(C_6_H_5_)_4_, and Ge(C_6_H_5_)_4_ crystals in air are 1.10, 1.26, and 0.46 s, respectively. *Φ*_p_(RT) of C(C_6_H_5_)_4_, Si(C_6_H_5_)_4_, and Ge(C_6_H_5_)_4_ crystals are 3.1%, 5.1%, and 17%, respectively. Analysis of the triplet yields of C(C_6_H_5_)_4_, Si(C_6_H_5_)_4_, and Ge(C_6_H_5_)_4_ in the crystalline state indicates that the appearance of persistent RTP is mostly driven by the large decrease of *k*_nr_(RT) +*k*_q_(RT) caused by crystallization. Analysis using vibrational spin–orbit coupling (VSOC) at RT indicates that the large decrease of *k*_nr_(RT) +*k*_q_(RT) is caused by not the decrease of *k*_nr_(RT) but the large decrease of *k*_q_(RT) owing to the small diffusion of triplet excitons at RT. Quantum chemical calculations reveal that the small diffusion of triplet excitons is caused by the small overlap between the highest occupied molecular orbitals (HOMOs), which originates from the localization of the HOMOs over the two phenylene rings induced by the π degeneracy of the steric and highly symmetric conjugated structures. For Ge(C_6_H_5_)_4_, the HOMO localization in the highly symmetric conjugated structures also induce moderate charge‐transfer (CT) character in transitions between high‐order singlet excited states (S*_m_*) and S_0_. Stronger spin–orbit coupling (SOC) between the moderate CT characteristics of the S*_m_*–S_0_ transitions and local excited (LE) characteristics of the T_1_–S_0_ transition contribute to the large increase of *k*_p_ independent of *k*_nr_(RT). This knowledge of electronic characteristics, *k*_p_, *k*_nr_(RT), and *k*_q_(RT) of conjugated molecular crystals will be important for realizing efficient persistent RTP from conjugated molecular crystals.

## 2 Results and Discussion

### 2.1 Emission Characteristics

**Figure 1:**
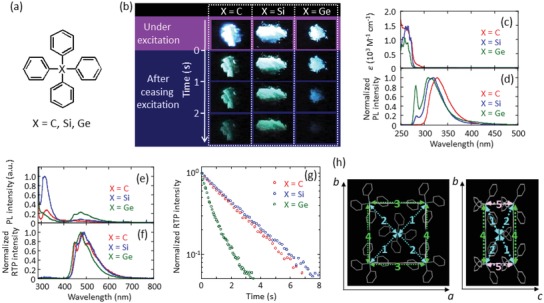
Optical characteristics and crystalline structures of C(C_6_H_5_)_4_, Si(C_6_H_5_)_4_, and Ge(C_6_H_5_)_4_. a) Chemical structures of C(C_6_H_5_)_4_, Si(C_6_H_5_)_4_, and Ge(C_6_H_5_)_4_. b) Changes in the luminescence of the three crystals under excitation at 280 nm and after ceasing excitation. c) Absorption and d) fluorescence spectra in THF at RT. In (d), the peak at 280 nm is caused by scattering of excitation light and the emission intensity was normalized to 1. Emission spectra of the crystals e) under excitation at 280 nm at RT and f) after ceasing excitation. In (e), the rapid increase below 300 nm is caused by scattering of the excitation light from the crystals. In (f), emission intensity was normalized to 1. g) RT emission decay characteristics of the crystals at 490 nm after ceasing excitation. h) Crystalline structures of Ge(C_6_H_5_)_4_ at RT.

The chemical structures of C(C_6_H_5_)_4_, Si(C_6_H_5_)_4_, and Ge(C_6_H_5_)_4_ are shown in **Figure** **1**a. Figure 1b shows the emission behavior of the crystals upon excitation at 280 nm and after ceasing excitation. The crystals exhibited blue emission upon excitation and then blue–green emission remained after ceasing excitation. To discuss the emission characteristics of C(C_6_H_5_)_4_, Si(C_6_H_5_)_4_, and Ge(C_6_H_5_)_4_, their absorption and emission spectra were measured in tetrahydrofuran (THF) and the crystalline state. In THF, the three compounds absorbed light with an energy higher than 300 nm (Figure 1c) and emitted ultraviolet fluorescence from 300 to 400 nm (Figure 1d). The fluorescence spectrum shifted to slightly higher energy with increasing atomic weight of center atom (X) of C(C_6_H_5_)_4_, Si(C_6_H_5_)_4_, and Ge(C_6_H_5_)_4_ crystals. Time‐dependent density functional theory (TDDFT) calculations also showed a small blueshift of the transition from S_0_ to the lowest singlet excited state (S_1_) with increasing atomic weight of X (Table S1, Supporting Information). The fluorescence quantum yields at RT (*Φ*_f_(RT)) of C(C_6_H_5_)_4_, Si(C_6_H_5_)_4_, and Ge(C_6_H_5_)_4_ in THF were 31%, 18%, and 2.7%, respectively. Because the fluorescence lifetimes at RT (τ_f_(RT)) of C(C_6_H_5_)_4_, Si(C_6_H_5_)_4_, and Ge(C_6_H_5_)_4_ were 4.0, 4.4, and 2.7 ns, respectively, the rate constants of fluorescence (*k*_f_) of C(C_6_H_5_)_4_, Si(C_6_H_5_)_4_, and Ge(C_6_H_5_)_4_ determined using *k*_f_ = *Φ*_f_(RT)τ_f_(RT) were 7.7 × 10^7^, 4.0 × 10^7^, and 9.9 × 10^6^ s^−1^, respectively.

Figure 1e shows the emission spectra of C(C_6_H_5_)_4_, Si(C_6_H_5_)_4_, and Ge(C_6_H_5_)_4_ crystals upon excitation at 280 nm in air. C(C_6_H_5_)_4_ and Si(C_6_H_5_)_4_ crystals showed distinct emission from 290 to 400 nm, whereas Ge(C_6_H_5_)_4_ exhibited a very weak emission peak in this wavelength range along with a tail from the scattering of the excitation light at 280 nm. The emission from 290 to 400 nm was designated as fluorescence because of its decay lifetime of several nanoseconds. The *k*_f_ values of C(C_6_H_5_)_4_, Si(C_6_H_5_)_4_, and Ge(C_6_H_5_)_4_ crystals determined from *Φ*_f_(RT) and τ_f_(RT) were 7.3 × 10^7^, 2.3 × 10^7^, and 8.8 × 10^6^ s^−1^, respectively, which were similar rates to those observed for isolated C(C_6_H_5_)_4_, Si(C_6_H_5_)_4_, and Ge(C_6_H_5_)_4_ molecules in THF. The fluorescence spectrum of the crystals shifted to slightly higher energy with increasing atomic weight of X and TDDFT calculations also showed this tendency (Table S2, Supporting Information). The similar fluorescence energy, fluorescence spectral shape, and *k*_f_ before and after crystallization suggest that the MOs related to fluorescence of the three compounds did not interact strongly between molecules in the crystalline structures. In addition, this indicates that the transition dipoles related to the S_1_–S_0_ transition of each aromatic molecule did not interact strongly in the crystalline structure.

A large difference between the isolated aromatics in solution and aromatic crystals is the appearance of RTP. Figure 1c also shows that a small emission peak appeared from 400 to 700 nm under excitation at 280 nm. This emission peak was assigned to RTP because it remained for a long time after the excitation was ceased, as reported by Clapp.^9^ Figure 1f shows normalized RTP spectra of the crystals after ceasing excitation at 280 nm. The spectral shape of the emission from the three crystals was comparable with that of the phosphorescence spectra of isolated C(C_6_H_5_)_4_, Si(C_6_H_5_)_4_, and Ge(C_6_H_5_)_4_ in 2‐methyl THF frozen at 77 K (Figure S1, Supporting Information). Similar RTP or T_1_ energies for the three isolated molecules as well as those of the crystals were also calculated by TD‐DFT (Table S1 and S2, Supporting Information). The average τ_p_(RT) values determined for the C(C_6_H_5_)_4_, Si(C_6_H_5_)_4_, and Ge(C_6_H_5_)_4_ crystals in air were 1.10, 1.26, and 0.45 s, respectively (Figure 1g). *Φ*_p_(RT) of the C(C_6_H_5_)_4_, Si(C_6_H_5_)_4_, and Ge(C_6_H_5_)_4_ crystals in air were 3.1%, 5.1%, and 17%, respectively. We carefully measured *Φ*_p_(RT) and τ_p_(RT) because triplet accumulation by strong excitation intensity triggers fluorescence resonance energy transfer from S_1_ to accumulated triplet excitons, phosphorescence resonance energy transfer from T_1_ to accumulated triplet excitons, and triplet–triplet annihilation, which cause underestimation of *Φ*_p_(RT) and τ_p_(RT).^12^, ^60^, ^61^ We confirmed that underestimation did not occur when 280 nm excitation light with a power of 1.0 mW cm^−2^ was used because linear increases of fluorescence and RTP were observed around this excitation intensity (Figure S2, Supporting Information). Although there are a few heavy atom‐free conjugated crystals with *Φ*_p_(RT) > 10%, we note that *Φ*_p_(RT) of C(C_6_H_5_)_4_ and Si(C_6_H_5_)_4_ were comparable with those of the recently reported crystallization‐induced persistent RTP materials and *Φ*_p_(RT) of the Ge(C_6_H_5_)_4_ crystals was much higher than those of many other conjugated molecular crystals with τ_p_(RT) of ≈0.5 s.^57^, ^58^

### 2.2 Physical Parameters to Investigate the Generation and Deactivation of Triplet Excitons

To understand the difference of *Φ*_p_(RT) for the three crystals, intrinsic physical parameters relating RTP can be determined using the quantum yield of phosphorescence at temperature *T* (*Φ*_p_(*T*)) and the phosphorescence lifetime at *T* (τ_p_(*T*)) as shown in the following equations^12^, ^58^, ^62^

(1) $$\Phi_{p}\left(T\right)=\Phi_{\mathrm{isc}}\left(T\right)k_{p}/\left(k_{p}+k_{\mathrm{nr}}\left(T\right)+k_{q}\left(T\right)\right)$$

(2) $$\tau_{p}\left(T\right)=1/\left(k_{p}+k_{\mathrm{nr}}\left(T\right)+k_{q}\left(T\right)\right)$$

where *Φ*_isc_(*T*) is the quantum yield of the triplet yield at *T* and *k*_q_(*T*) is the quenching rate caused by oxygen and quenching at *T* K because of trap states and surface traps after triplet exciton migration. To intrinsically investigate the origin of the appearance of persistent RTP, the determination of *Φ*_isc_(RT), *k*_p_, *k*_nr_(RT), and *k*_q_(RT) is necessary. However, previous discussions of *Φ*_isc_(RT), *k*_p_, *k*_nr_(RT), and *k*_q_(RT) of heavy atom‐free molecules contained numerous assumptions and were phenomenological for the persistent RTP of molecular aggregates. Therefore, a photophysical platform to determine and interpret *Φ*_isc_(RT), *k*_p_, *k*_nr_(RT), and *k*_q_(RT) of heavy atom‐free molecular aggregates is crucial for researchers to design state‐of‐the‐art molecular functions for the ultralong‐lived RT triplet excitons generated by a variety of heavy‐atom free conjugated structures.

### 2.3 Investigation of *Φ*_isc_(RT) by Comparison of Experimentally Observed and Theoretical *k*_p_

To investigate the origin of the appearance of the RTP characteristics in the crystals, *Φ*_isc_(RT) of the three types of crystals was estimated. Although *Φ*_isc_(RT) may often be different between dispersed conjugated molecules and an aggregate of the same molecules because of the change of *k*_f_ and the rate constant of intersystem crossing (ISC) between S_1_ and T_1_ at RT (*k*_isc_(RT)), no experimental methods to quantify *Φ*_isc_(RT) of crystalline materials have yet been developed. In this paper, we estimated *Φ*_isc_(RT) by assuming *Φ*_isc_(RT) ≈ 1 − *Φ*_f_(RT).^18^ It is considered that this approximation is generally applicable when there is no conical intersection between S_0_ and S_1_ in addition to a large energy gap (>2.17 eV) between S_1_ and S_0_ in the structure after relaxation from the Franck–Condon excited state. *Φ*_isc_(RT) of C(C_6_H_5_)_4_, Si(C_6_H_5_)_4_, and Ge(C_6_H_5_)_4_ crystals estimated based on *Φ*_isc_(RT) ≈ 1 − *Φ*_f_(RT) were 95%, 81%, and 97%, respectively. Then, *Φ*_isc_(RT), *Φ*_p_(RT), and τ_p_(RT) were substituted into Equation (3) to determine *k*_p_

(3) $$\Phi_{p}\left(\mathrm{RT}\right)=\Phi_{\mathrm{isc}}\left(\mathrm{RT}\right)k_{p}\tau_{p}\left(\mathrm{RT}\right)$$

The calculated *k*_p_ values of C(C_6_H_5_)_4_, Si(C_6_H_5_)_4_, and Ge(C_6_H_5_)_4_ were 3.0 × 10^−2^, 5.0 × 10^−2^, and 3.9 × 10^−1^ s^−1^, respectively.

To check the validity of the estimated *k*_p_ values based on *Φ*_isc_(RT) ≈ 1 − *Φ*_f_(RT), the experimentally determined *k*_p_ were compared with *k*_p_ obtained by quantum chemical calculations. Recently, *k*_p_ of dispersed heavy atom‐free conjugated structures in a highly rigid amorphous matrix was predicted well by TDDFT with SOC which can be included as a perturbation based on the scalar relativistic orbitals (pSOC‐TDDFT).^56^ Therefore, the same method was used to calculate *k*_p_ of the three crystals. X‐ray diffraction (XRD) analysis revealed that the three crystals possessed body‐centered tetragonal lattices with five kinds of dimers (dimer 1, dimer 2, dimer 3, dimer 4, and dimer 5) in each crystalline structure at RT, as shown in Figure 1h. When the triplet energies of the monomer and dimer 1–5 were calculated without changing the conformation determined by XRD analysis at RT, each of dimer 1–5 had eight triplet states of comparable energy with that of T_1_ and other triplet states that were much higher in energy than T_1_ (Table S3, Supporting Information). When an *i*‐order triplet state with different energy contained in dimer 1–5 is defined as T*_i_*, dimer 1–5 have 40 triplet states with comparable energy. Because each crystalline lattice of the C(C_6_H_5_)_4_, Si(C_6_H_5_)_4_, and Ge(C_6_H_5_)_4_ crystals contains the same number of dimer 1–5, *k*_p_ can be calculated as the average value based on Boltzmann distribution of T*_i_*–S_0_ transitions (*i* = 1–40) using Equation (4), 63

(4) $$k_{p}\;\;=\;\;\left[\Sigma_{i}k_{\mathrm{pi}}\exp\left(-\Delta E_{T_{1}-T_{i}}/kT\right)\right]/\Sigma_{i}\exp\left(-\Delta E_{T_{1}-T_{i}}/kT\right)$$

where $\Delta E_{T_{1}{\mathrm{–T}}_{i}}$ is the energy difference between T_1_ and T*_i_*, and *k*_pi_ is the rate constant of phosphorescence from T*_i_* to S_0_, which was determined by a quantum chemical calculation. In the quantum chemical calculation, configurations confirmed using XRD at RT were used to calculate physical parameters. To calculate *k*_p_ of the dimers, the SOC operator within the zeroth‐order regular approximation was used as the operator for SOC ($\bar{H_{\mathrm{SO}}}$) and *k*_p_ was treated as a perturbation based on the scalar relativistic orbitals. Hybrid‐B3LYP and TZP were used as exchange‐correlation functionals and the Slater‐type all‐electron basis set, respectively. *k*_p_ of C(C_6_H_5_)_4_, Si(C_6_H_5_)_4_, and Ge(C_6_H_5_)_4_ calculated from Equation (4) using the configurations of dimer 1–5 were 4.7 × 10^−2^, 6.4 × 10^−2^, and 4.5 × 10^−1^ s^−1^, respectively. The calculated *k*_p_ values are comparable with the experimentally estimated ones assuming *Φ*_isc_(RT) ≈ 1 − *Φ*_f_(RT) (**Table** **1**). Therefore, *Φ*_isc_(RT) ≈ 1 − *Φ*_f_(RT) is applicable to the three types of crystals and C(C_6_H_5_)_4_, Si(C_6_H_5_)_4_, and Ge(C_6_H_5_)_4_ crystals are considered to display large *Φ*_isc_(RT). Although we calculated *k*_p_ using the monomer structures of C(C_6_H_5_)_4_, Si(C_6_H_5_)_4_, and Ge(C_6_H_5_)_4_, the values were overestimated (Table 1). This suggests that T_1_ is delocalized in dimers in the crystals. Quantum chemical calculations of the rate constant of ISC from S_1_ to T_1_ suggested that the very large *Φ*_isc_(RT) is not caused by crystallization‐induced enhancement of *Φ*_isc_(RT) (Figure S3, Supporting Information). Because *Φ*_isc_(RT) approaches 100% for the three types of crystals, analysis of all of *k*_p_, *k*_nr_(RT), and *k*_q_(RT) is important to understand the origin of persistent RTP from these crystals under ambient conditions.

**Table 1:** Photophysical parameters of C(C_6_H_5_)_4_, Si(C_6_H_5_)_4_, and Ge(C_6_H_5_)_4_

Compound	Solution		Solid								
	Exp.^a)^	Calc.	Exp.							Calc.	
	*k*_f_	*k*_p_ ^b)^, ^c)^	*Φ*_f_[RT]	*k*_f_	*Φ*_p_[RT]	τ_p_[RT]	*k*_p_ ^d)^	*k*_nr_[RT]^d)^	*k*_q_[RT]^d)^	*k*_p_ ^b)^, ^e)^	VSOC[RT]^b)^, ^f)^
	[s^−1^]	[s^−1^]	[%]	[s^−1^]	[%]	[s]	[s^−1^]	[s^−1^]	[s^−1^]	[s^−1^]	[a.u.]
C(C_6_H_5_)_4_ ^g)^	7.7 × 10^7^	8.9 × 10^−2^	5.1	7.3 × 10^7^	3.1	1.10	3.0 × 10^−2^	5.0 × 10^−1^	3.6 × 10^−1^	4.7 × 10^−2^	–
Si(C_6_H_5_)_4_ ^g)^	4.0 × 10^7^	1.3 × 10^−1^	18.6	2.3 × 10^7^	5.1	1.26	5.0 × 10^−2^	5.2 × 10^−1^	2.6 × 10^−1^	6.4 × 10^−2^	4.7 × 10^2^
Ge(C_6_H_5_)_4_ ^g)^	9.9 × 10^6^	9.6 × 10^−1^	2.8	8.8 × 10^6^	17.0	0.45	3.9 × 10^−1^	1.6 × 10^0^	3.5 × 10^−1^	4.5 × 10^−1^	9.0 × 10^2^
Chrysene^h)^	–	3.5 × 10^−3^	16.0	–	0.91	1.36	8.0 × 10^−3^	5.7 × 10^−1^	1.6 × 10^−1^	–	1.0 × 10^2^

^a)^ In THF solution

^b)^ Information about SOC was treated as a perturbation based on the scalar relativistic orbitals. Hybrid‐B3LYP and TZP were used as exchange‐correlation functionals and the Slater‐type all‐electron basis set, respectively

^c)^ Calculated using monomer structure

^d)^ Calculated using τ_p_(RT) and *Φ*_isc_(RT) = 1 −*Φ*_f_(RT)

^e)^ Calculated as average value of dimers 1–5 determined by XRD

^f)^ VSOC(RT) means $P_{p}\left(RT\right){\left|\partial 〈\Psi_{0}^{1\left(0\right)}|\bar{H_{SO}}|\Psi_{1}^{3\left(0\right)}〉/\partial Q_{p}\right|}^{2}$. Conformations including vibration modes of monomer were optimized at T_1_ using density functional theory (Gaussian09/B3LYP/6‐31G(d))

^g)^ Data for solids were measured in the crystalline state

^h)^ Data for solids were measured for 0.3 wt% chrysene‐doped β‐estradiol in the amorphous state.

### 2.4 Investigation of the Origin of the Small *k*_q_(RT) Including Separation of *k*_nr_(RT) and *k*_q_(RT)

**Figure 2:**
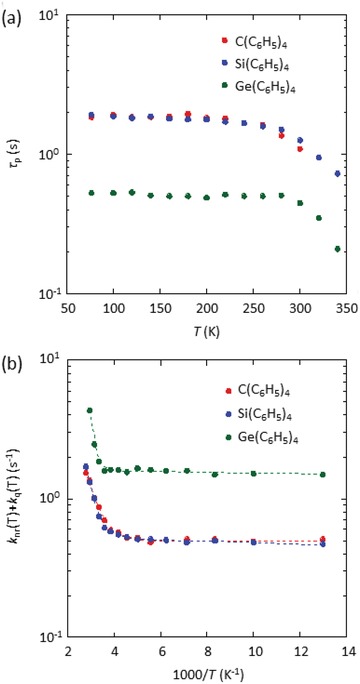
Temperature dependence of the phosphorescence characteristics of C(C_6_H_5_)_4_, Si(C_6_H_5_)_4_, and Ge(C_6_H_5_)_4_ crystals. a) τ_p_(*T*). b) *k*_nr_(*T*) + *k*_q_(*T*). Dashed lines are fitted with a sum of two exponential functions.

To discuss *k*_nr_(RT) and *k*_q_(RT) of the crystals, the temperature dependence of τ_p_(*T* ) was measured, as shown in **Figure** **2**a. τ_p_(*T* ) of C(C_6_H_5_)_4_, Si(C_6_H_5_)_4_, and Ge(C_6_H_5_)_4_ crystals hardly decreased from 77 K to RT. By substituting experimentally determined *k*_p_ (Table 1) into Equation (2), the Arrhenius plots of *k*_nr_(*T*) + *k*_q_(*T*) for the crystals were constructed, as presented in Figure 2b. Each curve could be fitted using a sum of two exponential functions. The increase of *k*_nr_(*T* ) + *k*_q_(*T* ) at higher temperature has been ascribed to the increase of *k*_q_(*T* ), which is caused by quenching of triplet excitons at defects after triplet exciton migration.^59^ The activation energy of triplet exciton migration is theoretically expressed as the sum of the reorganization energies of hole and electron transfer.^64^ The reorganizations induced by hole and electron transfer have large configuration changes compared with those caused by atomic vibrations in a molecule and large reorganizations typically have high activation energies. Therefore, the larger increase of *k*_nr_(*T* ) + *k*_q_(*T* ) at higher temperature in Figure 2b was caused by the increase of *k*_q_(*T* ) triggered by triplet exciton migration and the smaller increase of *k*_nr_(*T*) + *k*_q_(*T*) at lower temperature was caused by the increase of *k*_nr_(*T*) depending on the intramolecular vibrations of chromophores.^12^, ^56^, ^58^, ^59^, ^65^, ^66^ By using an exponential function at higher temperature for each crystal, *k*_q_(RT) of C(C_6_H_5_)_4_, Si(C_6_H_5_)_4_, and Ge(C_6_H_5_)_4_ crystals were quantified as 3.6 × 10^−1^, 2.6 × 10^−1^, and 3.5 × 10^−1^ s^−1^, respectively. Thus, very small and comparable quenching of triplet excitons at RT was observed for the three crystals.

Recently, indeed cooperative analysis of the diffusion length of triplet excitons at RT (*L*_T_(RT)) using a microscopic technique and estimation of *L*_T_(RT) using a quantum chemical calculation verified that *k*_q_(RT) observed at higher temperature was mainly affected by quenching of triplet excitons at trap sites after triplet exciton migration.^59^ In this analysis, it was considered that *k*_q_(RT) was proportional to the diffusion constant of triplet excitons at RT (*D*_T_(RT)) when crystals had comparable defect densities for triplet excitons. *D*_T_(RT) is generally expressed as

(5) $$D_{T}\left(\mathrm{RT}\right)=L_{T}{\left(\mathrm{RT}\right)}^{2}/\tau_{p}\left(\mathrm{RT}\right)$$

**Figure 3:**
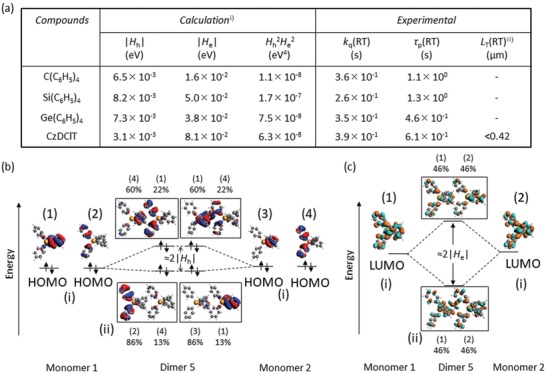
Comparison of calculated physical parameters related to diffusion of triplet excitons and illustration of the difference of hole and electron transfer integrals using molecular orbitals. a) Summary of |*H*_h_| and |*H*_e_| of a representative dimer showing the largest *H*_h_^2^ *H*_e_^2^ and experimentally observed *k*_q_(RT), τ_p_(RT), and *L*_T_(RT) in air of C(C_6_H_5_)_4_, Si(C_6_H_5_)_4_, and Ge(C_6_H_5_)_4_. i) Conformations in crystalline structures determined by XRD at RT are used. GGA:PW91 and TZP were used as exchange‐correlation functionals and the Slater‐type all‐electron basis, respectively, to calculate |*H*_h_| and |*H*_e_|. Data of dimer 3 for C(C_6_H_5_)_4_. Data of dimer 5 for Si(C_6_H_5_)_4_ and Ge(C_6_H_5_)_4_. ii) The value is determined using microscope in ref. 59. b) Structures of molecular orbitals causing the small |*H*_h_| of dimer 5 in the Ge(C_6_H_5_)_4_ crystalline lattice. c) Structures of molecular orbitals related to the large |*H*_e_| of dimer 5 in the Ge(C_6_H_5_)_4_ crystalline lattice.

To experimentally obtain *L*_T_(RT), a 2D RTP pattern can be compared with the 2D pattern of excitation light obtained when the excitation light is focused on a single crystal.^59^ However, the analysis of C(C_6_H_5_)_4_, Si(C_6_H_5_)_4_, and Ge(C_6_H_5_)_4_ single crystals requires high‐resolution focusing of excitation light below 320 nm using an objective lens with large numerical aperture (NA); such light is not able to penetrate commercially available objective lenses. Although excitation by two‐photon absorption using nanosecond laser pulses at 355 nm was attempted, no persistent RTP signal was obtained from C(C_6_H_5_)_4_, Si(C_6_H_5_)_4_, and Ge(C_6_H_5_)_4_ crystals. In the previous approach, however, *D*_T_(RT) was well estimated from the transfer integrals of holes and electrons related to triplet excitons by quantum chemical calculations using crystalline structures. *D*_T_(RT) can be expressed based on the concept of double charge transfer as follows^64^

(6) $$D_{T}\left(\mathrm{RT}\right)\propto H_{e}{}^{2}H_{h}{}^{2}$$

where *H*_e_ is the transfer integral of lowest unoccupied molecular orbitals (LUMOs) and *H*_h_ is the transfer integral of HOMOs. Therefore, *H*_e_^2^ *H*_h_^2^ of C(C_6_H_5_)_4_, Si(C_6_H_5_)_4_, and Ge(C_6_H_5_)_4_ crystals was investigated. Because the *H*_e_^2^ *H*_h_^2^ value of dimer 5 shown in Figure 1h was much larger than those of dimer 1–4 for Si(C_6_H_5_)_4_ and Ge(C_6_H_5_)_4_ crystals (Table S4, Supporting Information), the *H*_e_^2^ *H*_h_^2^ value of dimer 5 is important to discuss triplet exciton diffusion in Si(C_6_H_5_)_4_ and Ge(C_6_H_5_)_4_ crystals. For the C(C_6_H_5_)_4_ crystal, dimer 2 had the largest value of *H*_e_^2^ *H*_h_^2^. The highest *H*_e_^2^ *H*_h_^2^ values for C(C_6_H_5_)_4_, Si(C_6_H_5_)_4_, and Ge(C_6_H_5_)_4_ crystals were 1.1 × 10^−8^, 1.7 × 10^−7^, and 7.5 × 10^−8^ eV^4^, respectively (**Figure** **3**a). In our previous report, 9‐(3,5‐dichlorophenyl)‐9*H*‐carbazole (CzDClT) crystals with τ_p_(RT) of 0.61 s exhibited very small *D*_T_(RT) (3 × 10^−9^ cm^2^ s^−1^) and *L*_T_(RT) (<0.42 µm), as shown in Figure 3a.^59^ This report showed that the largest *H*_e_^2^ *H*_h_^2^ value of a dimer along the *b*‐axis of CzDClT crystals was 6.3 × 10^−8^ eV^4^ (Figure 3a). That is, CzDClT, C(C_6_H_5_)_4_, Si(C_6_H_5_)_4_, and Ge(C_6_H_5_)_4_ crystals have comparable *H*_e_^2^ *H*_h_^2^. Therefore, it could be considered that C(C_6_H_5_)_4_, Si(C_6_H_5_)_4_, and Ge(C_6_H_5_)_4_ crystals have very small *L*_T_(RT), similar to CzDClT crystals, and the inefficient migration of triplet excitons in the three crystals results in the minimization of *k*_q_(RT).

For C(C_6_H_5_)_4_, Si(C_6_H_5_)_4_, and Ge(C_6_H_5_)_4_ crystals, it might be generally considered that the small *D*_T_(RT) is caused by the large twisted aromatic structures. However, we note that |*H*_e_| of dimer 5 of Si(C_6_H_5_)_4_ and Ge(C_6_H_5_)_4_ are comparable to that of dimers in a rubrene single crystal (4.7 × 10^−2^ eV), which has very large *D*_T_(RT) (Figure 3a).^67^ Compared with the large |*H*_e_|, dimer 5 of Si(C_6_H_5_)_4_ and Ge(C_6_H_5_)_4_ have much smaller |*H*_h_|. Therefore, small overlap between the HOMOs of the monomers in dimer 5 causes the small *L*_T_(RT) and results in the small *k*_q_(RT) below 10^0^ s^−1^. Figure 3b presents the relationship between the HOMOs of the two monomers (monomer 1 and 2) in dimer 5 of a Ge(C_6_H_5_)_4_ crystal. Each monomer had two comparable MOs corresponding to HOMOs ((i) of Figure 3b). Interestingly, the HOMOs of both monomer 1 and 2 were not delocalized over all four phenylene rings but were instead localized over two phenylene rings. The localization of a HOMO over only two phenylene rings was caused by the degeneracy of the highly symmetric four‐phenylene rings of C(C_6_H_5_)_4_, Si(C_6_H_5_)_4_, and Ge(C_6_H_5_)_4_ structure.^68^ Because the two monomers were located such that the overlap of their HOMOs was minimized ((ii) of Figure 3b), HOMOs with very small splitting energy, approximately corresponding to 2|*H*_h_|, were obtained. Small |*H*_h_| based on the same mechanism were also determined for the Si(C_6_H_5_)_4_ and C(C_6_H_5_)_4_ crystals (Figure S4a,b, Supporting Information). For electron transfer, the LUMOs of monomer 1 and 2 were delocalized over all four phenylene rings ((i) in Figure 3c). Consequently, the large overlap of the LUMOs leads to large |*H*_e_|, as visually observed for dimer 5 of a Ge(C_6_H_5_)_4_ crystal ((ii) in Figure 3c) and Si(C_6_H_5_)_4_ and C(C_6_H_5_)_4_ crystals (Figure S4c,d, Supporting Information respectively). Although the MOs of a triphenylsilane molecule as an asymmetric structure were checked, its HOMO and LUMO were both delocalized over the whole molecule because of the lack of π degeneracy, causing large overlap of HOMOs between molecules, which facilitates triplet exciton migration (Figure S5, Supporting Information). We note that 2D highly symmetric structures often have large overlap of MOs related to triplet exciton diffusion because of the effective stacking of planar conjugated structures, although each monomer has two kinds of moderately separated HOMOs caused by the π degeneracy (Figure S6, Supporting Information). Therefore, steric hindrance and a highly symmetric structure may be important to achieve very small overlap of MOs related to triplet exciton diffusion despite their close molecular packing in crystalline structures. Although the above discussion is based on the assumption that triplet excitons are localized in monomers, in Section 2.2. the estimation of *k*_p_ by quantum chemical calculations was more accurate when triplet excitons were delocalized over dimers (Table 1). We note that analysis regarding the transfer integrals between two dimers contained in crystalline structure also indicates that the localization of HOMOs caused by the steric and highly symmetric structures in C(C_6_H_5_)_4_, Si(C_6_H_5_)_4_, and Ge(C_6_H_5_)_4_ triggers inefficient hole transfer (Section 3 and Figure S7 in the Supporting Information), which contributes to the suppressed triplet exciton diffusion and leads to the minimization of *k*_q_(RT).

### 2.5 Investigation of *k*_nr_(RT) for the Three Types of Crystals

**Figure 4:**
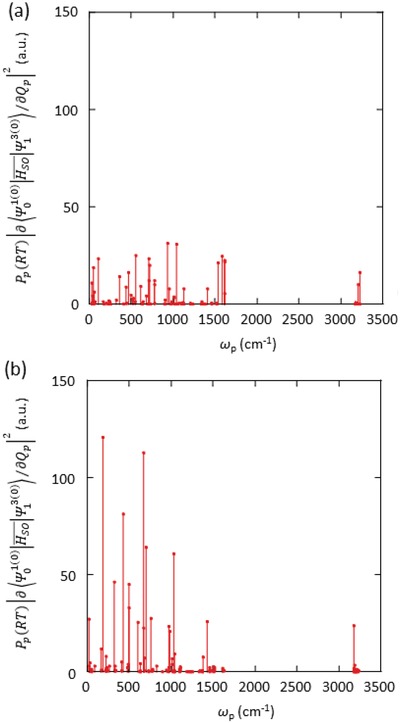
Relationship between $P_{p}\left(\mathrm{RT}\right){\left|\partial \left\langle \Psi_{0}^{1\left(0\right)}|\bar{H_{\mathrm{SO}}}|\Psi_{1}^{3\left(0\right)}\right\rangle /\partial Q_{p}\right|}^{2}$ and ω_p_ for a) Si(C_6_H_5_)_4_ and b) Ge(C_6_H_5_)_4_ monomers. Conformations including normal vibration modes were optimized at T_1_ using density functional theory (Gaussian09/B3LYP/6‐31G(d)). SOC data were treated as perturbations based on the scalar relativistic orbitals. Hybrid‐B3LYP and TZP were used as exchange‐correlation functionals and the Slater‐type all‐electron basis set, respectively.

**Figure 5:**
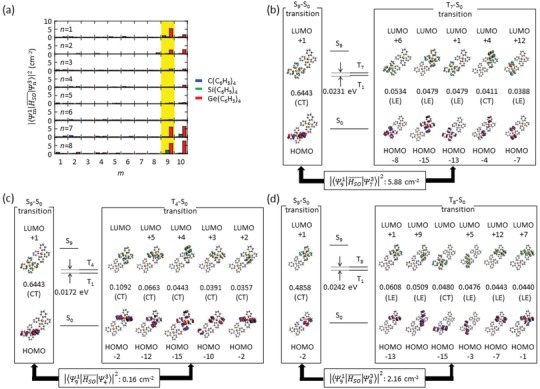
SOC between S*_m_* and T*_n_* (*n* = 1–8) and MOs involved in the SOC of dimer 2 of C(C_6_H_5_)_4_, Si(C_6_H_5_)_4_, and Ge(C_6_H_5_)_4_ crystals. a) Relationship between ${\left|\left\langle \Psi_{n}^{1}|\bar{H_{SO}}|\Psi_{n}^{3}\right\rangle \right|}^{2}$ and *m* for dimer 2 of C(C_6_H_5_)_4_, Si(C_6_H_5_)_4_, and Ge(C_6_H_5_)_4_. b) MOs related to the S_9_–S_0_ and T_7_–S_0_ transitions of dimer 2 of Ge(C_6_H_5_)_4_. c) MOs related to the S_9_–S_0_ and T_4_–S_0_ transitions of dimer 2 of Ge(C_6_H_5_)_4_. d) MOs related to the S_9_–S_0_ and T_8_–S_0_ transitions of dimer 2 of Si(C_6_H_5_)_4_.

The separation of *k*_q_(RT) based on the discussion of the small triplet exciton diffusion length of the C(C_6_H_5_)_4_, Si(C_6_H_5_)_4_, and Ge(C_6_H_5_)_4_ crystals allows us to quantify *k*_nr_(RT) of the three types of crystals. The small increase of *k*_nr_(*T*) + *k*_q_(*T*) from 77 to 260 K is caused by vibration‐based nonradiative deactivation from T_1_. Using the exponential fitting line at lower temperature in Figure 2b, *k*_nr_(RT) of C(C_6_H_5_)_4_, Si(C_6_H_5_)_4_, and Ge(C_6_H_5_)_4_ crystals were quantified as 5.0 × 10^−1^, 5.2 × 10^−1^, and 1.6 × 10^0^ s^−1^, respectively. Thus, *k*_nr_(RT) of Ge(C_6_H_5_)_4_ crystals is 3.2 and 3.1 times larger than those of C(C_6_H_5_)_4_ and Si(C_6_H_5_)_4_ crystals, respectively. Using theoretical equations containing a VSOC term explained in the 1970s,^69^, ^70^ *k*_nr_(RT) of a variety of dispersed heavy atom‐free conjugated molecules have recently been predicted using VSOC considering vibrational factors at RT. In these calculations, dispersed heavy atom‐free conjugated molecules with comparable T_1_ energy could be approximately predicted using $\Sigma P_{p}\left(RT\right){\left|\partial \left\langle \Psi_{0}^{1\left(0\right)}|\bar{H_{SO}}|\Psi_{1}^{3\left(0\right)}\right\rangle /\partial Q_{p}\right|}^{2}$, where $\Psi_{0}^{1(0)}$ is the wavefunction of S_0_ with normal vibrations, $\Psi_{1}^{3(0)}$ is the wavefunction of T_1_ with normal vibrations, *H*_SO_ is the Hamiltonian operator related to SOC, *P*_p_(RT) is the vibrational factor at RT, and *Q*_p_ is the coordinate of atoms.^56^ **Figure** **4** illustrates the relationship between $P_{p}\left(RT\right){\left|\partial \left\langle \Psi_{0}^{1\left(0\right)}|\bar{H_{SO}}|\Psi_{1}^{3\left(0\right)}\right\rangle /\partial Q_{p}\right|}^{2}$ and wavenumber (ω_p_) for Si(C_6_H_5_)_4_ and Ge(C_6_H_5_)_4_ crystals. By integrating the signals in **Figure** **5**, $\Sigma P_{p}\left(RT\right){\left|\partial \left\langle \Psi_{0}^{1\left(0\right)}|\bar{H_{SO}}|\Psi_{1}^{3\left(0\right)}\right\rangle /\partial Q_{p}\right|}^{2}$ of Si(C_6_H_5_)_4_ and Ge(C_6_H_5_)_4_ crystals were determined to be 4.7 × 10^2^ and 9.0 × 10^2^ a.u, respectively. We previously reported that nonaggregated chrysene showing green T_1_ energy has *k*_nr_(RT) of 5 × 10^−1^ s^−1^ in rigid short conjugated amorphous media and $\Sigma P_{p}\left(RT\right){\left|\partial \left\langle \Psi_{0}^{1\left(0\right)}|\bar{H_{SO}}|\Psi_{1}^{3\left(0\right)}\right\rangle /\partial Q_{p}\right|}^{2}$ of chrysene was calculated to be 1.0 × 10^2^ a.u. (Table 1).^56^ By considering the ratios of $\Sigma P_{p}\left(\mathrm{RT}\right){\left|\partial \left\langle \Psi_{0}^{1\left(0\right)}|\bar{H_{\mathrm{SO}}}|\Psi_{1}^{3\left(0\right)}\right\rangle /\partial Q_{p}\right|}^{2}$ for Si(C_6_H_5_)_4_, Ge(C_6_H_5_)_4_, and chrysene as well as *k*_nr_(RT) = 5 × 10^−1^ s^−1^ for dispersed chrysene, *k*_nr_(RT) of Si(C_6_H_5_)_4_ and Ge(C_6_H_5_)_4_ crystals were estimated to increase up to 2.5 × 10^0^ and 4.5 × 10^0^ s^−1^, respectively, if the molecules freely vibrate in the crystalline structures. However, experimentally observed *k*_nr_(RT) of Si(C_6_H_5_)_4_ and Ge(C_6_H_5_)_4_ crystals were less than half of the estimated values. Therefore, some vibrations of Si(C_6_H_5_)_4_ and Ge(C_6_H_5_)_4_ molecules in the crystals are slightly suppressed compared with those of an isolated molecule. The large decrease of the nonradiative deactivation from T_1_ after crystallization is often attributed to the restriction of the molecular vibrations of the conjugated molecules in the crystal lattice. However, we note that at least for C(C_6_H_5_)_4_, Si(C_6_H_5_)_4_, and Ge(C_6_H_5_)_4_ crystals, the decrease is mostly caused by the decrease of *k*_q_(RT) and the vibrations of molecules in the crystalline state are not strongly related to the large decrease of nonradiative deactivation from T_1_ at RT. Although the emission spectrum becomes very sharp if the vibrations are largely suppressed, the lack of sharp features observed for the C(C_6_H_5_)_4_, Si(C_6_H_5_)_4_, and Ge(C_6_H_5_)_4_ crystals also indicates the weak suppression of molecular vibrations in the crystalline structure. The appearance of persistent RTP from Si(C_6_H_5_)_4_ doped in Zeonex under high vacuum conditions also supports this conclusion (Figure S8, Supporting Information).

The discussion in Section 2.4 and 2.5 indicates that quenching caused by triplet exciton diffusion is the main reason for the accelerated nonradiative deactivation from T_1_ at RT for C(C_6_H_5_)_4_, Si(C_6_H_5_)_4_, and Ge(C_6_H_5_)_4_ crystals and molecular vibrations hardly affect triplet deactivation. Therefore, control of electronic structures to minimize triplet exciton migration at RT in dense conjugated molecular packing is a feasible approach to obtain ultralong‐lived T_1_ at RT to realize persistent RTP characteristics from heavy atom‐free conjugated molecular crystals. Although it is unclear if this is the case for all recently reported aromatic crystals, our previous report using a recent representative aromatic crystal with persistent RTP characteristics and this report investigating an old example of nonpolar aromatic crystals with persistent RTP characteristics suggest that this mechanism is applicable to a variety of aromatic crystals.

### 2.6 Discussion of the Large Difference of *k*_p_ between the Three Types of Crystals

The suppression of the migration of triplet excitons at RT contributes to the appearance of ultralong‐lived RT triplet excitons of the C(C_6_H_5_)_4_, Si(C_6_H_5_)_4_, and C(C_6_H_5_)_4_ crystals. Meanwhile, *k*_p_ > *k*_nr_(RT) is necessary to obtain more efficient persistent RTP characteristics. Ge(C_6_H_5_)_4_ crystals show much larger *Φ*_p_(RT) compared with those of Si(C_6_H_5_)_4_ and C(C_6_H_5_)_4_ crystals. Table 1 reveals that *k*_nr_(RT) of the Ge(C_6_H_5_)_4_ crystals is 3.1 times higher than that of the Si(C_6_H_5_)_4_ crystals and *k*_p_ of Ge(C_6_H_5_)_4_ is 7.6 times larger than that of Si(C_6_H_5_)_4_. Although this causes the much larger *Φ*_p_(RT) of Ge(C_6_H_5_)_4_ compared with those of the other types of crystals, the reason for the increase of *k*_p_ independent of *k*_nr_(RT) in conjugated crystalline materials is still unclear for conjugated molecular crystals. To discuss this point, physical factors related to *k*_p_ were investigated for the three crystals.

Recently, good predictions of *k*_p_ of several dispersed heavy atom‐free aromatic structures using pSOC‐TDDFT have been reported.^56^ For each dimer 1–5 of C(C_6_H_5_)_4_, Si(C_6_H_5_)_4_, and Ge(C_6_H_5_)_4_ crystals, *k*_p_ was approximated using the following equations^56^, ^71^

(7) $$k_{p}\propto \sum_{m}{\left(\mu_{S_{m}\to S_{0}}\lambda_{m}\right)}^{2}$$

(8) $$\lambda_{m}\approx \frac{\sum_{n}\frac{\left|\left\langle \Psi_{m}^{1}|\bar{H_{\mathrm{SO}}}|\Psi_{n}^{3}\right\rangle \right|}{\left|\Delta E_{S_{m}-T_{n}}\right|}\exp\left(-\frac{\Delta E_{T_{n}-T_{1}}}{kT}\right)}{\sum_{n}\exp\left(-\frac{\Delta E_{T_{n}-T_{1}}}{kT}\right)}\;,\;(n=1-8)$$

where $\mu_{S_{m}\to S_{0}}$ is the transition dipole moment from S*_m_* to S_0_, $\Psi_{m}^{1}$ is the wavefunction of S*_m_*, $\Psi_{n}^{3}$ is the wavefunction of T*_n_*, $\Delta E_{S_{m}-T_{n}}$ is the energy difference between S*_m_* and T*_n_*, and $\Delta E_{T_{n}-T_{1}}$ is the energy difference between T*_n_* and T_1_. Again, because each dimer has eight triplet states with comparable energy (*n* = 1–8), λ_m_ in Equation (8) was considered as the average value for *n* = 1–8. Equations (7) and (8) indicate that large $\mu_{S_{m}\to S_{0}}{\;}^{2}$, large ${\left|\left\langle \Psi_{m}^{1}|\bar{H_{\mathrm{SO}}}|\Psi_{n}^{3}\right\rangle \right|}^{2}$, and small ${\left|\Delta E_{S_{m}-T_{n}}\right|}^{2}$ approximately lead to large *k*_p_. Because a large difference was observed between *k*_p_ of Si(C_6_H_5_)_4_ and Ge(C_6_H_5_)_4_ crystals, the following three paragraphs discuss which of $\mu_{S_{m}\to S_{0}}{\;}^{2}$, ${\left|\left\langle \Psi_{m}^{1}|\bar{H_{\mathrm{SO}}}|\Psi_{n}^{3}\right\rangle \right|}^{2}$, and ${\left|\Delta E_{S_{m}-T_{n}}\right|}^{2}$ contributes the most to the enhancement of *k*_p_ for Ge(C_6_H_5_)_4_ crystals.

Moderate CT characteristics of the S*_m_*–S_0_ transitions and strong LE characteristics of the T*_n_*–S_0_ transitions (*n* = 1–8) increase ${\left|\left\langle \Psi_{m}^{1}|\bar{H_{SO}}|\Psi_{n}^{3}\right\rangle \right|}^{2}$. Figure 5a displays the relationship between ${\left|\left\langle \Psi_{m}^{1}|\bar{H_{SO}}|\Psi_{n}^{3}\right\rangle \right|}^{2}$ and *m* of dimer 2 for C(C_6_H_5_)_4_, Si(C_6_H_5_)_4_, and Ge(C_6_H_5_)_4_ crystals. When ${\left|\left\langle \Psi_{9}^{1}|\bar{H_{SO}}|\Psi_{n}^{3}\right\rangle \right|}^{2}$ of Ge(C_6_H_5_)_4_ crystals is focused (yellow background of Figure 5a), ${\left|\left\langle \Psi_{9}^{1}|\bar{H_{SO}}|\Psi_{1}^{3}\right\rangle \right|}^{2}$, ${\left|\left\langle \Psi_{9}^{1}|\bar{H_{SO}}|\Psi_{7}^{3}\right\rangle \right|}^{2}$, and ${\left|\left\langle \Psi_{9}^{1}|\bar{H_{SO}}|\Psi_{8}^{3}\right\rangle \right|}^{2}$ are large whereas other ${\left|\left\langle \Psi_{9}^{1}|\bar{H_{SO}}|\Psi_{n}^{3}\right\rangle \right|}^{2}$ are very small. Figure 5b presents MOs related to ${\left|\left\langle \Psi_{9}^{1}|\bar{H_{SO}}|\Psi_{7}^{3}\right\rangle \right|}^{2}$ of dimer 2 of Ge(C_6_H_5_)_4_ crystals. In the S_9_–S_0_ transition, an electron is delocalized over four phenylene rings in S_0_ and partly localized over two phenylene rings in S_9_. Therefore, the S_9_–S_0_ transition has moderate CT character. Thus, moderate CT characteristics are caused by the localization of the HOMOs of dimer 5 over two phenylene rings. The localization of HOMOs in dimer 5 is also caused by the localization of the HOMO of each monomer over two phenylene rings, as explained above (see Figure 3b). Unlike the moderate CT character of the S_9_–S_0_ transition, the T_7_–S_0_ transition has multiple LE characteristics. Because SOC between CT and LE characteristics is large according to the El‐Sayed rule,^72^ this contributes to the large increase of ${\left|\left\langle \Psi_{9}^{1}|\bar{H_{SO}}|\Psi_{7}^{3}\right\rangle \right|}^{2}$. Figure 5c depicts MOs related to ${\left|\left\langle \Psi_{9}^{1}|\bar{H_{SO}}|\Psi_{4}^{3}\right\rangle \right|}^{2}$ and the T_4_–S_0_ transition has multiple CT characteristics. The similar CT characteristics of the S_9_–S_0_ and T_4_–S_0_ transitions will cause small ${\left|\left\langle \Psi_{9}^{1}|\bar{H_{SO}}|\Psi_{4}^{3}\right\rangle \right|}^{2}$ according to the El‐Sayed rule. Checking the character of other transitions revealed that S*_m_*–S_0_ transitions always have CT character and T*_n_*–S_0_ transitions (*n* = 1–8) have multiple LE transition characteristics when large ${\left|\left\langle \Psi_{m}^{1}|\bar{H_{SO}}|\Psi_{n}^{3}\right\rangle \right|}^{2}$ is obtained (Figure S9, Supporting Information). Indeed, ${\left|\left\langle \Psi_{m}^{1}|\bar{H_{SO}}|\Psi_{n}^{3}\right\rangle \right|}^{2}$ becomes small when a S*_m_*–S_0_ transition has CT character and T*_n_*–S_0_ transition (*n* = 1–8) is mainly composed of multiple CT transitions (Figure S10a–c, Supporting Information). In addition, we noticed that ${\left|\left\langle \Psi_{m}^{1}|\bar{H_{SO}}|\Psi_{n}^{3}\right\rangle \right|}^{2}$ becomes small when an S*_m_*–S_0_ transition has mixed CT and LE characteristics (Figure S10d, Supporting Information). The mixture of multiple transition characteristics potentially lessens the difference between the characteristics of S*_m_*–S_0_ and T*_n_*–S_0_ transitions. This tendency was also observed for other dimers of Ge(C_6_H_5_)_4_. Therefore, π degeneracy caused by highly symmetric structure induces moderate CT characteristics in S*_m_*, which contributes to large ${\left|\left\langle \Psi_{m}^{1}|\bar{H_{SO}}|\Psi_{n}^{3}\right\rangle \right|}^{2}$ and leads to the increase of *k*_p_.

We note that heavy atom effect accelerated *k*_p_ but did not contribute to the increase of *Φ*_p_(RT). Figure 5d shows MOs related to ${\left|\left\langle \Psi_{9}^{1}|\bar{H_{SO}}|\Psi_{8}^{3}\right\rangle \right|}^{2}$ of dimer 2 of Si(C_6_H_5_)_4_. The S_9_–S_0_ transition has moderate CT character that is similar to the characteristics of the S_9_–S_0_ transition of dimer 2 of Ge(C_6_H_5_)_4_ crystals. Furthermore, the T_8_–S_0_ transition of dimer 2 of Si(C_6_H_5_)_4_ contains strong LE characteristics (Figure 5d), which is also similar to the T_7_–S_0_ transition of dimer 2 of Ge(C_6_H_5_)_4_ crystals (Figure 5b). Therefore, values of SOC are considered to be comparable if the Ge atom of Ge(C_6_H_5_)_4_ does not affect the SOC. However, ${\left|\left\langle \Psi_{9}^{1}|\bar{H_{SO}}|\Psi_{7}^{3}\right\rangle \right|}^{2}$of dimer 2 of Ge(C_6_H_5_)_4_ crystals (5.88 cm^−2^) is 2.7 times larger than ${\left|\left\langle \Psi_{9}^{1}|\bar{H_{SO}}|\Psi_{8}^{3}\right\rangle \right|}^{2}$ of dimer 2 of Si(C_6_H_5_)_4_ crystals (2.16 cm^−2^). Thus, the large positive charge of the Ge nucleus in |*H*_so_| also partly contributed to the increase of ${\left|\left\langle \Psi_{m}^{1}|\bar{H_{SO}}|\Psi_{n}^{3}\right\rangle \right|}^{2}$ of the Ge(C_6_H_5_)_4_ crystals. However, the experimentally quantified *k*_nr_(RT) of Ge(C_6_H_5_)_4_ crystals was also 3.1 times larger than that of Si(C_6_H_5_)_4_ crystals. The similar values can be reasonably explained by the heavy atom effect increasing *k*_nr_(RT) and *k*_p_ by the same magnitude. However, the enhancement of *k*_p_ of Ge(C_6_H_5_)_4_ crystals compared with that of Si(C_6_H_5_)_4_ crystals is larger than is the case for *k*_nr_(RT).

The Ge(C_6_H_5_)_4_ crystal had many moderate CT states at S*_m_* compared with the situations for the Si(C_6_H_5_)_4_ and C(C_6_H_5_)_4_ crystals, which mainly contributed to the increase of *Φ*_p_(RT). The number of S*_m_*–T*_n_* (*n* = 1–8) transitions with ${\left|\left\langle \Psi_{m}^{1}|\bar{H_{SO}}|\Psi_{n}^{3}\right\rangle \right|}^{2}$ >5.1 cm^−2^ for dimer 2 was counted for the Ge(C_6_H_5_)_4_ crystal. Because the contribution of the heavy atom effect to the increase of ${\left|\left\langle \Psi_{m}^{1}|\bar{H_{SO}}|\Psi_{n}^{3}\right\rangle \right|}^{2}$ increased by ≈3 times upon moving from Si(C_6_H_5_)_4_ to Ge(C_6_H_5_)_4_, the number of S*_m_*–T*_n_* (*n* = 1–8) transitions with ${\left|\left\langle \Psi_{m}^{1}|\bar{H_{SO}}|\Psi_{n}^{3}\right\rangle \right|}^{2}$ >1.7 cm^−2^, where 1.7 (5.1/3) was used to exclude the contribution of the heavy atom effect to ${\left|\left\langle \Psi_{m}^{1}|\bar{H_{SO}}|\Psi_{n}^{3}\right\rangle \right|}^{2}$, was determined. Ge(C_6_H_5_)_4_ had five kinds of S*_m_*–T*_n_* transitions with ${\left|\left\langle \Psi_{m}^{1}|\bar{H_{SO}}|\Psi_{n}^{3}\right\rangle \right|}^{2}$ > 5.1 cm^−2^ for dimer 2 whereas dimer 2 of Si(C_6_H_5_)_4_ had only two kinds of S*_m_*–T*_n_* transitions with ${\left|\left\langle \Psi_{m}^{1}|\bar{H_{SO}}|\Psi_{n}^{3}\right\rangle \right|}^{2}$ > 1.7 cm^−2^ (Table S5, Supporting Information). This tendency was also observed for other dimers (Tables S6–S8, Supporting Information). Because the increase of ${\left|\left\langle \Psi_{m}^{1}|\bar{H_{SO}}|\Psi_{n}^{3}\right\rangle \right|}^{2}$ can increase *k*_p_ independent of *k*_nr_(RT), the formation of many states with moderate CT characteristics in S*_m_* is an origin of the large *Φ*_p_(RT) of Ge(C_6_H_5_)_4_.

**Figure 6:**
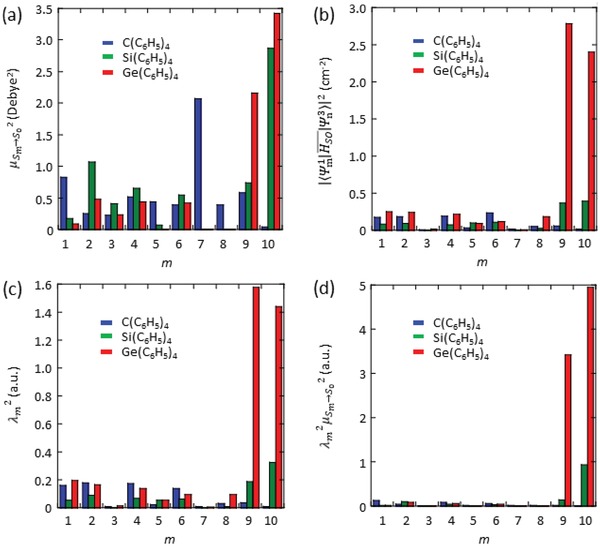
Relationships between photophysical parameters related to *k*_p_ and *m* of dimer 2 in C(C_6_H_5_)_4_, Si(C_6_H_5_)_4_, and Ge(C_6_H_5_)_4_ crystals. Relationships between a) *m* and μSm→S02, b) *m* and ${\left|\left\langle \Psi_{n}^{1}|\bar{H_{SO}}|\Psi_{n}^{3}\right\rangle \right|}^{2}$ averaged for *n* = 1–8, c) *m* and λ_m_^2^ averaged for *n* = 1–8, and d) *m* and $\mu_{S_{m}\to S_{0}}{\;}^{2}\lambda_{m}{\;}^{2}$ averaged for *n* = 1–8.

In Equations (7) and (8), the increase of ${\left|\left\langle \Psi_{m}^{1}|\bar{H_{SO}}|\Psi_{n}^{3}\right\rangle \right|}^{2}$ mainly contributed to the large enhancement of *k*_p_ of Ge(C_6_H_5_)_4_ crystals compared with those of C(C_6_H_5_)_4_ and Si(C_6_H_5_)_4_ crystals, whereas the other two factors ($\mu_{S_{m}\to S_{0}}$ and |$\Delta E_{S_{m}-T_{n}}$|) did not contribute to *k*_p_. **Figure** **6**a presents the $\mu_{S_{m}\to S_{0}}$ for dimer 2 of C(C_6_H_5_)_4_, Si(C_6_H_5_)_4_, and Ge(C_6_H_5_)_4_ crystals, which were shown in Table S9 (Supporting Information). The sum of $\mu_{S_{m}\to S_{0}}{\;}^{2}$ for all *m* (Σ*_m_* $\mu_{S_{m}\to S_{0}}{}^{2}$) is proportional to *k*_p_, as shown in Equation (7). Figure 6a indicates Σ*_m_* $\mu_{S_{m}\to S_{0}}{}^{2}$ were similar for the three types of crystals because the ratio of Σ*_m_* $\mu_{S_{m}\to S_{0}}{}^{2}$ of C(C_6_H_5_)_4_, Si(C_6_H_5_)_4_, and Ge(C_6_H_5_)_4_ crystals was 1.00:1.14:1.26. Therefore, $\mu_{S_{m}\to S_{0}}{}^{2}$ hardly contributes to the enhancement of *k*_p_ in the Ge(C_6_H_5_)_4_ crystals. Figure 6b shows the average of ${\left|\left\langle \Psi_{m}^{1}|\bar{H_{SO}}|\Psi_{n}^{3}\right\rangle \right|}^{2}$ for *n* of dimer 2 of C(C_6_H_5_)_4_, Si(C_6_H_5_)_4_, and Ge(C_6_H_5_)_4_ crystals, which were determined using data provided in Table S5 (Supporting Information). The increase of ${\left|\left\langle \Psi_{m}^{1}|\bar{H_{SO}}|\Psi_{n}^{3}\right\rangle \right|}^{2}$ of Ge(C_6_H_5_)_4_ crystals compared with those of C(C_6_H_5_)_4_ and Si(C_6_H_5_)_4_ crystals in the cases of *m* = 9 and 10 strongly contributed to the enhancement of *k*_p_ of the Ge(C_6_H_5_)_4_ crystals. Figure 6c presents the average λ_m_^2^ for all *n* of dimer 2, which were calculated using Equation (8). The shape of the distribution of the dependence of λ_m_^2^ on *m* (Figure 6c) was similar to that of ${\left|\left\langle \Psi_{m}^{1}|\bar{H_{SO}}|\Psi_{n}^{3}\right\rangle \right|}^{2}$ on *m* (Figure 6b). Because Equation (8) indicates that λ_m_ depends on ${\left|\left\langle \Psi_{m}^{1}|\bar{H_{SO}}|\Psi_{n}^{3}\right\rangle \right|}^{2}$ and |$\Delta E_{S_{m}-T_{n}}$|, the similar distribution shapes in Figure 6b,c indicate that |$\Delta E_{S_{m}-T_{n}}$| does not cause the difference of *k*_p_. Figure 6d shows the average of μSm→S02λ_m_^2^ for all *n* of dimer 2. Because the two distinct peaks at *m* = 9 and 10 is observed in Figure 6d is similar to that in Figure 6b, the large ${\left|\left\langle \Psi_{m}^{1}|\bar{H_{SO}}|\Psi_{n}^{3}\right\rangle \right|}^{2}$ of *m* = 9 and 10 accelerates *k*_p_ for Ge(C_6_H_5_)_4_ crystals. The overall tendencies of dimer 2 presented in Figure 6a–d were also observed for the other dimers. In addition, the relationship between Figure 6a–d was observed when μSm→S02, ${\left|\left\langle \Psi_{m}^{1}|\bar{H_{SO}}|\Psi_{n}^{3}\right\rangle \right|}^{2}$, λ_m_^2^, and μSm→S02λ_m_^2^ of dimer 1–5 were integrated (Figure S11, Supporting Information). Therefore, the large ${\left|\left\langle \Psi_{m}^{1}|\bar{H_{SO}}|\Psi_{n}^{3}\right\rangle \right|}^{2}$ for *m* = 9 and 10 accelerates *k*_p_ of the Ge(C_6_H_5_)_4_ crystals. Although it may be considered that the high‐order excited state energies such as S_9_ and S_10_ are too high, we note that the S_9_–S_0_ and S_10_–S_0_ energies are not much larger than the S_1_–S_0_ energy (Table S10, Supporting Information). Therefore, the moderate CT characteristics of S*_m_*–S_0_ transitions induced by degeneracy of HOMOs caused by highly symmetric π structures in addition to LE characteristics of the T_1_–S_0_ transition contribute to large ${\left|\left\langle \Psi_{m}^{1}|\bar{H_{SO}}|\Psi_{n}^{3}\right\rangle \right|}^{2}$, which mainly causes the increase of *k*_p_ independent of *k*_nr_(RT). This is an origin of the more efficient persistent RTP of Ge(C_6_H_5_)_4_ crystals compared with that of C(C_6_H_5_)_4_ and Si(C_6_H_5_)_4_ crystals.

### 2.7 Roles of Local Electronic Structure in Achieving Efficient Persistent RTP

This investigation of *k*_p_, *k*_nr_(RT), and *k*_q_(RT) considering the electronic structures of heavy atom‐free aromatic crystals may provide us smooth strategies to explore highly efficient persistent RTP.

The suppression of the migration of triplet excitons at RT is necessary to minimize *k*_q_(RT) to realize large τ_p_(RT) in crystals of heavy atom‐free conjugated molecules. Small overlap of MOs related to hole and/or electron transfer between dimers in a crystalline structure is a way to obtain such suppression. A previous report^59^ revealed that CT conjugated molecules show strongly localized MOs, which sometimes minimizes the overlap of MOs and suppresses triplet exciton migration. This report proposes that the localization of MOs utilizing the π degeneracy of steric and highly symmetric conjugated structures is another way to sometimes minimize *k*_q_(RT) caused by triplet exciton diffusion. To suppress triplet exciton migration, effective separation of the HOMO and LUMO is necessary. However, the localization of both HOMO and LUMO is not suitable for persistent RTP because it causes large separation of HOMO and LUMO. Such separation results in rapid delayed fluorescence without persistent emission through rapid reverse ISC from T_1_ to S_1_ as a result of the very small energy difference between S_1_ and T_1_. Strong CT characteristics of the T_1_–S_0_ transition generally increase $\left\langle \Psi_{0}^{1\left(0\right)}|\bar{H_{SO}}|\Psi_{1}^{3\left(0\right)}\right\rangle$, as seen for benzophenone.^18^, ^56^ Because this also increases the magnitude of the change of $\left\langle \Psi_{0}^{1\left(0\right)}|\bar{H_{SO}}|\Psi_{1}^{3\left(0\right)}\right\rangle$ depending on the change of coordinates of atoms (*Q*_p_), i.e., VSOC, the CT characteristics of the T_1_–S_0_ transition strongly increase *k*_nr_(RT). Conversely, $\left\langle \Psi_{0}^{1\left(0\right)}|\bar{H_{SO}}|\Psi_{1}^{3\left(0\right)}\right\rangle$ becomes small and VSOC also decreases when all triplet states near T_1_ possess LE characteristics, resulting in small *k*_nr_(RT). Even when the HOMO or LUMO is strongly localized, the T_1_–S_0_ transition still has moderate LE characteristics. Therefore, conjugated structures with a HOMO or LUMO that is strongly localized are candidate molecules for persistent RTP. When the T_1_–S_0_ transition has LE character, introduction of moderate CT characteristics of the S*_m_*–S_0_ transition is effective. This report proposes that the localized MOs in some highly symmetric conjugated structures induce moderate CT characteristics in S*_m_*–S_0_ transitions.

From the above discussion, molecules with electronic structures that show strong localization of MOs involved in the T_1_–S_0_ transition potentially contribute to the increase of *k*_p_ independent of *k*_nr_(RT) as well as minimization of *k*_q_(RT) to achieve efficient persistent RTP. However, appropriate molecular packing so that the localized MOs are separated is also crucial. Although prediction of crystalline packing is desirable, it is still very difficult. However, exploring molecular structures with strongly localized MOs is a way to increase the percentage of MOs with small overlap to minimize *k*_q_(RT). Thus, steric and highly symmetric conjugated structures in addition to moderate CT conjugated structures substituted with electron‐donating or ‐accepting substituents are candidates to meet the requirement of strongly localized MOs involved in the T_1_–S_0_ transition. Because the electronic structure of a monomer can be predicted using current quantum calculations, prescreening of electronic structures may provide an approach to increase the percentage of prepared heavy atom‐free conjugated crystals displaying efficient persistent RTP.

## 3 Conclusions

We examined the efficient persistent RTP from nonpolar and highly symmetric C(C_6_H_5_)_4_, Si(C_6_H_5_)_4_, and Ge(C_6_H_5_)_4_ crystals in air. *Φ*_p_(RT) of C(C_6_H_5_)_4_, Si(C_6_H_5_)_4_, and Ge(C_6_H_5_)_4_ crystals in air were 3.1%, 5.1%, and 17%, respectively. τ_p_(RT) of C(C_6_H_5_)_4_, Si(C_6_H_5_)_4_, and Ge(C_6_H_5_)_4_ crystals in air were 1.10, 1.26, and 0.45 s, respectively. The appearance of crystallization‐induced persistent RTP in air was caused by the large decrease of *k*_nr_(RT) + *k*_q_(RT) upon crystallization rather than the increase of *Φ*_isc_(RT). Quantum chemical calculations of factors related to Dexter type‐electron exchange and the temperature dependence of *k*_nr_(T) + *k*_q_(T) indicated that the small *k*_nr_(RT) + *k*_q_(RT) is caused by the minimization of *k*_q_(RT) originating from the small diffusion length of triplet excitons in the crystals at RT. The small triplet diffusion length could be explained by inefficient hole transfer caused by the small overlap of HOMOs originating from HOMO localization, which is induced by the π degeneracy of the steric and highly symmetric molecular structure. The temperature dependence of *k*_nr_(RT) + *k*_q_(RT) also indicated that *k*_nr_(RT) of C(C_6_H_5_)_4_, Si(C_6_H_5_)_4_, and Ge(C_6_H_5_)_4_ crystals are small and do not contribute much to the decrease of *k*_nr_(RT) + *k*_q_(RT). Analysis using VSOC considering vibration at RT supported the small contribution of *k*_nr_(RT) to the decrease of *k*_nr_(RT) + *k*_q_(RT). While the increase of *k*_nr_(RT) of Ge(C_6_H_5_)_4_ crystals was small compared with that of Si(C_6_H_5_)_4_ crystals, *k*_p_ of Ge(C_6_H_5_)_4_ crystals was 7.6 times larger than that of Si(C_6_H_5_)_4_ crystals, which caused the large *Φ*_p_(RT) of Ge(C_6_H_5_)_4_ crystals. The large enhancement of *k*_p_ independent of the increase of *k*_nr_(RT) is mainly caused by the increase of SOC between the moderate CT characteristics of S*_m_*–S_0_ transitions and T_1_–S_0_ transition with LE character. The moderate CT characteristics of S*_m_*–S_0_ transitions are induced by the HOMO localization in the highly symmetric conjugated structure of Ge(C_6_H_5_)_4_. Thus, the localized HOMOs in highly symmetric conjugated structures play roles in acceleration of *k*_p_ independent of *k*_nr_(RT) as well as minimization of *k*_q_(RT).

In the last five years, a variety of heavy atom‐free conjugated molecular crystals with persistent RTP have been reported. Our investigation indicates that persistent RTP of molecular crystals with small *Φ*_p_(RT), like C(C_6_H_5_)_4_ and Si(C_6_H_5_)_4_ crystals, is obtained through small *k*_q_(RT) by suppression of triplet exciton migration at RT. Conversely, Ge(C_6_H_5_)_4_ crystals showed increased *k*_p_ independent of *k*_nr_(RT) in addition to the large decrease of *k*_q_(RT). This is first overall analysis of *k*_p_, *k*_nr_(RT), and *k*_q_(RT) of heavy atom‐free conjugated molecular aggregates. The high simultaneous correlation between experimentally observed *k*_p_, *k*_nr_(RT), and *k*_q_(RT) and those estimated from quantum chemical calculations suggests that a procedure to evaluate triplet excitons of heavy atom‐free molecular aggregates is feasible. Because new aromatic crystals showing persistent RTP and moderate *Φ*_p_(RT) have been reported recently, design of electronic structures that lead to *k*_p_ > *k*_nr_(RT) without increase of *k*_nr_(RT) is crucial to obtain highly efficient persistent RTP from heavy atom‐free conjugated molecules under ambient conditions. Pre‐screening using quantum chemical calculations focusing on moderate CT character of S*_m_* and the localization of HOMO or LUMO will help researchers to find suitable electronic structures and materials. The enhancement of the characteristics of singlet and triplet states is important for developing new applications using persistent RT emission.

## 4 Experimental Section

C(C_6_H_5_)_4,_ Si(C_6_H_5_)_4_, and Ge(C_6_H_5_)_4_ powders were purified as Section 1 in the Supporting Information. XRD measurements of C(C_6_H_5_)_4_, Si(C_6_H_5_)_4_, and Ge(C_6_H_5_)_4_ single crystals were performed at RT using a Bruker SMART APEX II ULTRA/CCD diffractometer. Absorption spectra of the samples were measured by an absorption spectrometer (V‐760, Jasco International Co., Ltd., Japan). Fluorescence and persistent RTP spectra and τ_p_(T) were measured using a photonic multichannel analyzer (PMA‐12, Hamamatsu Photonics, Japan) as a photodetector and an excitation unit of a fluorometer (FP‐8300, Jasco International Co., Ltd.) as an excitation source. Persistent RTP spectra were collected by detecting emission spectra soon after ceasing excitation. *Φ*_f_(RT) and *Φ*_p_(RT) were determined using an absolute luminescence quantum yield measurement system (C9920‐02G, Hamamatsu Photonics). The method described in the Supporting Information of an earlier study was used to determine *Φ*_p_(RT).^12^ The temperature used to determine the temperature dependence of τ_p_(RT) was controlled by a cryostat (Optistat‐DNV, Oxford, United Kingdom). Fluorescence lifetime at RT was measured with a fluorescence lifetime spectrometer (C11367, Hamamatsu Photonics).

For calculations of *k*_p_ and VSOC, The SOC operator within the zeroth‐order regular approximation (ZORA) was $\bar{H_{\mathrm{SO}}}$ and the parameter $\left\langle \Psi_{m}^{1\left(0\right)}|\bar{H_{SO}}|\Psi_{1}^{3\left(0\right)}\right\rangle$ was treated as a perturbation based on the scalar relativistic orbitals. Values of ${\left|\left\langle \Psi_{m}^{1}|\bar{\left(H_{SO}\right)}|\Psi_{n}^{3}\right\rangle \right|}^{2}$ were expressed as sum of data between one singlet state and three kinds of triplet states with comparable excited energy. For quantum calculations of *k*_p_, the molecular configurations that were determined by XRD of single crystals were used without structure optimization. Each *k*_p_ was treated as a perturbation based on the scalar relativistic orbitals. Hybrid‐B3LYP and TZP were used as exchange‐correlation functionals and the Slater‐type all‐electron basis set, respectively. To calculate VSOC, conformations including normal mode vibrations were optimized at T_1_ using density functional theory (Gaussian09/B3LYP/6‐31G(d)). Information about the SOC depending on each vibration in the conformations was treated as a perturbation based on the scalar relativistic orbitals. Hybrid‐B3LYP and TZP were used in VSOC calculations as exchange‐correlation functionals and the Slater‐type all‐electron basis set, respectively. $P_{p}\left(RT\right){\left|\partial \left\langle \Psi_{0}^{1\left(0\right)}|\bar{H_{SO}}|\Psi_{1}^{3\left(0\right)}\right\rangle /\partial Q_{p}\right|}^{2}$ at each vibration was approximated according to the procedures reported in ref. 56. To calculate transfer integrals, the molecular configurations that were determined by XRD of single crystals were used without structure optimization. GGA‐PW91 and TZP were used as exchange‐correlation functionals and the Slater‐type all‐electron basis set, respectively.

## Conflict of Interest

The author declares no conflict of interest.

## Supporting information

Supplementary
