# Supplementary material for: Roles of Localized Electronic Structures Caused by π Degeneracy Due to Highly Symmetric Heavy Atom‐Free Conjugated Molecular Crystals Leading to Efficient Persistent Room‐Temperature Phosphorescence
Source: Adv Sci (Weinh). 2019 May 10;6(14):1900410. doi: 10.1002/advs.201900410 (PMC6661950; doi:10.1002/advs.201900410)
Supplement: Supplementary file 1 — Supplementary [file ADVS-6-1900410-s001.pdf]

Copyright WILEY-VCH Verlag GmbH & Co. KGaA, 69469 Weinheim, Germany, 2019.

ADVANCED SCIENCE
Open Access

Supporting Information

for *Adv. Sci.,* DOI: 10.1002/advs.201900410

Roles of Localized Electronic Structures Caused by π Degeneracy Due to Highly Symmetric Heavy Atom-Free Conjugated Molecular Crystals Leading to Efficient Persistent Room-Temperature Phosphorescence

*Shuzo Hirata**

Supporting Information

Roles of Localized Electric Structures caused by π Degeneracy due to Highly Symmetric Heavy Atom-free Conjugated Molecular Crystals leading to Efficient Persistent Room-temperature Phosphorescence

Shuzo Hirata[*]

Prof. S. Hirata
Department of Engineering Science, University of Electro-Communications, 1-5-1 Chofugaoka, Chofu, Tokyo 182-8585, Japan

E-mail: shuzohirata@uec.ac.jp

Contents

1. **Materials**
2. **Investigation of the rate constant of intersystem crossing from S_1 to T_1 using quantum chemical calculations**
3. **Analysis of the transfer integrals between two dimers contained in crystalline structure**
4. **Table S1-S10**
5. **Figure S1–S11**
6. **Supporting reference**

1. Materials

$C(C_6H_5)_4$, $Si(C_6H_5)_4$, and $Ge(C_6H_5)_4$ powders were purchased from Tokyo Chemical Industry Co., Ltd. Single crystals of $Si(C_6H_5)_4$ and $Ge(C_6H_5)_4$ were prepared by recrystallization from toluene solutions. The commercially available $C(C_6H_5)_4$ contained many impurities and did not form crystals well in recrystallization procedures. Therefore, single crystals of $C(C_6H_5)_4$ were obtained after purification by column chromatography using toluene/hexane and subsequent recrystallization from a toluene/hexane solution.

2. Investigation of the rate constant of intersystem crossing from S_1 to T_1 using quantum chemical calculations

The large Φ_{isc}(RT) of $C(C_6H_5)_4$, $Si(C_6H_5)_4$, and $Ge(C_6H_5)_4$ crystals, which were estimated from $\Phi_{isc}(RT)=1-\Phi_f(RT)$, were probably not caused by the crystalline-induced enhancement of Φ_{isc}(RT). k_{isc}(T) is generally expressed as,[S1]

$$k_{isc}(T) = \frac{2\pi}{\hbar}\sum_n |\langle \Psi_1^1|\overline{H_{SO}}|\Psi_n^3\rangle|^2\, exp\left[\frac{-(\lambda+\Delta E_{Tn-S1})^2}{4\lambda kT}\right]/\sqrt{4\lambda kT}, \qquad \text{(S1)}$$

where $\overline{H_{SO}}$ is the Hamiltonian operator related to SOC between S_1 and T_n, Ψ_1^1 is the wavefunction of S_1, Ψ_n^3 is the wavefunction of a high-order triplet excited state (T_n), λ is the reorganization energy for the intersystem crossing (ISC) from S_1 to T_n, $\Delta E_{S1\text{-}Tn}$ is the energy difference between T_n and S_1, and k is the Boltzmann constant. $|\langle \Psi_1^1|\overline{H_{SO}}|\Psi_n^3\rangle|^2$ of the monomers and dimer 1–5 for $C(C_6H_5)_4$, $Si(C_6H_5)_4$, and $Ge(C_6H_5)_4$ were separately calculated based on first-order perturbative SOC. Figure S3a–c shows the relationships between $|\langle \Psi_1^1|\overline{H_{SO}}|\Psi_n^3\rangle|^2$ and $\Delta E_{Tn\text{-}S1}$ for the monomers and dimer 1–5 of the three types of crystals. In equation S1, λ is generally between 0 and 0.5 eV.[S1] Therefore, the term $\exp(-(\lambda+\Delta E_{Tn\text{-}S1})^2/4\lambda kT)$ in Equation S1 becomes large when $\Delta E_{Tn\text{-}S1}$ is from −0.5 to 0 eV and $|\langle \Psi_1^1|\overline{H_{SO}}|\Psi_n^3\rangle|^2$ at $\Delta E_{Tn\text{-}S1}$=−0.5–0 eV mainly contributes to k_{isc}(RT). Therefore, investigation

of $|\langle\Psi_1^1|\overline{H_{SO}}|\Psi_n^3\rangle|^2$ at $\Delta E_{\text{Tn-S1}}$=−0.5–0 eV is important to discuss k_{isc}(RT) before and after crystallization. For the $C(C_6H_5)_4$ monomer, S_1–T_5 to S_1–T_{14} transitions involve ISC at $\Delta E_{\text{Tn-S1}}$=−0.5–0 eV, as shown in the yellow background of (i) in Figure S3a. The integration of $|\langle\Psi_1^1|\overline{H_{SO}}|\Psi_n^3\rangle|^2$ at $\Delta E_{\text{Tn-S1}}$=−0.5–0 eV was 2.85 cm^{-2} for the $C(C_6H_5)_4$ monomer. For dimer 1, 2, 3, 4, and 5 of the $C(C_6H_5)_4$ single crystal, the integrated values of $|\langle\Psi_1^1|\overline{H_{SO}}|\Psi_n^3\rangle|^2$ at $\Delta E_{\text{Tn-S1}}$=−0.5–0 eV were 2.87, 2.87, 2.75, 2.75, and 2.68 cm^{-2}, respectively (yellow background in Figure S3a). Therefore, $|\langle\Psi_1^1|\overline{H_{SO}}|\Psi_n^3\rangle|^2$ at $\Delta E_{\text{Tn-S1}}$=−0.5–0 eV does not change much among the monomer and dimer 1–5. Small differences of $|\langle\Psi_1^1|\overline{H_{SO}}|\Psi_n^3\rangle|^2$ at $\Delta E_{\text{Tn-S1}}$=−0.5–0 eV among the monomer and dimer 1–5 were also observed for $Si(C_6H_5)_4$ and $Ge(C_6H_5)_4$ (yellow background in Figure S3b and c, respectively). These findings indicate that k_{isc}(RT) of $C(C_6H_5)_4$, $Si(C_6H_5)_4$, and $Ge(C_6H_5)_4$ are not affected by crystallization.

3. Analysis of the transfer integrals between two dimers contained in crystalline structure

It has been reported that a rubrene single crystal shows efficient anisotropic triplet exciton migration at RT of over 3 μm along the *b*-axis of the crystalline structure.[59,67] Figure S7a(i) and (ii) depict the HOMO and LUMO in a rubrene monomer, respectively. The T_1–S_0 transition of rubrene involves a HOMO–LUMO transition. In a rubrene monomer, both the HOMO and LUMO are delocalized over a tetracene moiety. Because the HOMO and LUMO over the tetracene moiety overlap considerably in the dimer along the *b*-axis of a rubrene crystal, the HOMO and LUMO are delocalized over two tetracene moieties in a dimer (Figure S7a, (i) and (ii)). This causes substantial overlap of HOMOs and LUMOs of the two dimers in a tetramer along the *b*-axis of a rubrene crystal as well (Figure S7a, (iii)). Therefore, the large transfer integrals of holes and electrons in the two dimers explain the large triplet exciton

migration distance along the *b*-axis of a rubrene crystal. The calculated absolute value of the transfer integrals using the two dimers in a tetramer of rubrene were 3.6×10^{-2} eV for holes and 2.2×10^{-2} eV for electrons. On the other hand, the absolute value of the hole transfer integral calculated using dimer 5 of $Ge(C_6H_5)_4$ in a tetramer along the *c*-axis of a $Ge(C_6H_5)_4$ crystal was 1.7×10^{-3} eV. The small hole transfer integral in $Ge(C_6H_5)_4$ can be explained using a model in which triplet excitons are not delocalized all over dimer 5. Because each HOMO of a $Ge(C_6H_5)_4$ monomer is localized over two phenylene rings rather than four because of its highly symmetric structure, the HOMO of dimer 5 of $Ge(C_6H_5)_4$ is also not delocalized over all four phenylene rings (Figure S7b, (i)). Therefore, the localization of the HOMO of each monomer induces the localization of the HOMOs in dimer 5 for the $Ge(C_6H_5)_4$ structure. Consequently, the HOMOs of the two dimer 5 in a tetramer of $Ge(C_6H_5)_4$ do not overlap (Figure S7b, (iii)). This explains the small hole transfer integral of $Ge(C_6H_5)_4$ crystals. Conversely, the symmetric delocalization of the LUMO of each monomer over all phenylene rings causes delocalization of the LUMOs over all phenylene rings of dimer 5 (Figure S7b, (ii)). This leads to a large overlap between the LUMOs of the two dimer 5 in a tetramer along the *c*-axis of a $Ge(C_6H_5)_4$ crystal (Figure S7b, (iii)). Therefore, the large electron transfer integral of dimer 5 can be explained by a model in which triplet excitons are delocalized over two dimers. Indeed, the absolute value of the electron transfer integral calculated using dimer 5 of $Ge(C_6H_5)_4$ in a tetramer along the *c*-axis of a $Ge(C_6H_5)_4$ crystal was 2.4×10^{-2} eV, which is comparable to that of rubrene. Similar overall characteristics were also observed in pairs of other dimers of $Ge(C_6H_5)_4$ crystals and for the other two types of crystals. Therefore, the localization of HOMOs caused by the highly symmetric structures in $C(C_6H_5)_4$, $Si(C_6H_5)_4$, and $Ge(C_6H_5)_4$ triggers inefficient hole transfer, which contributes to the suppressed triplet exciton diffusion and leads to the minimization of k_q(RT).

4. Table S1-S10

Table S1. Calculated photophysical parameters of isolated $C(C_6H_5)_4$, $Si(C_6H_5)_4$, and $Ge(C_6H_5)_4$. Conformations were optimized using DFT (Gausian09/B3LYP/sdd) calculations. S_1 and T_1 energies and the oscillator strength for the S_0–S_1 transition (*f*) were determined by TD-DFT (Gausian09/B3LYP/sdd) calculations.

Compound	S_1		T_1		*f*
	[eV]	[nm]	[eV]	[nm]	
$C(C_6H_5)_4$	4.9793	249.03	3.5129	353.98	0.1490
$Si(C_6H_5)_4$	5.1952	238.68	3.6481	339.90	0.0029
$Ge(C_6H_5)_4$	5.2400	236.64	3.6632	338.50	0.0041

Table S2. Calculated photophysical parameters of $C(C_6H_5)_4$, $Si(C_6H_5)_4$, and $Ge(C_6H_5)_4$ in the crystalline state. Monomer configurations determined by single-crystal X-ray analyses were used to determine the S_1 and T_1 energies and oscillator strength (*f*) for the S_0–S_1 transition by TD-DFT (Gausian09/B3LYP/sdd) calculations.

Crystal	S_1		T_1		*f*
	[eV]	[nm]	[eV]	[nm]	
$C(C_6H_5)_4$	5.1386	241.31	3.8731	320.16	0.0136
$Si(C_6H_5)_4$	5.3742	230.73	3.9594	313.18	0.0006
$Ge(C_6H_5)_4$	5.4089	229.25	3.9639	312.82	0.0005

Table S3. k_{pn} and ΔE_{T1}-T_n values of $C(C_6H_5)_4$, $Si(C_6H_5)_4$, and $Ge(C_6H_5)_4$ crystals.

	$C(C_6H_5)_4$			$Si(C_6H_5)_4$			$Ge(C_6H_5)_4$		
i	k_{pn} (s^{-1})	ΔE_{T1}-T_i (eV)	Dimer type	k_{pi} (s^{-1})	ΔE_{T1}-T_i (eV)	Dimer type	k_{pi} (s^{-1})	ΔE_{T1}-T_i (eV)	Dimer type
1	0.0359	0.0000	Dimer 1	0.0333	0.0000	Dimer 5	0.3280	0.0000	Dimer 5
2	0.0361	0.0000	Dimer 2	0.0713	0.0016	Dimer 1	0.6270	0.0035	Dimer 1
3	0.0613	0.0025	Dimer 1	0.0728	0.0016	Dimer 2	0.6543	0.0035	Dimer 2
4	0.0614	0.0025	Dimer 2	0.0254	0.0033	Dimer 5	0.5160	0.0046	Dimer 1
5	0.0740	0.0040	Dimer 5	0.0233	0.0033	Dimer 5	0.5162	0.0046	Dimer 2
6	0.0216	0.0040	Dimer 5	0.0487	0.0039	Dimer 1	0.0061	0.0058	Dimer 3
7	0.1280	0.0043	Dimer 3	0.0475	0.0039	Dimer 2	0.0330	0.0058	Dimer 4
8	0.1282	0.0043	Dimer 4	0.0186	0.0059	Dimer 3	0.8660	0.0061	Dimer 3
9	0.0014	0.0043	Dimer 3	0.0165	0.0059	Dimer 4	0.8344	0.0061	Dimer 4
10	0.0013	0.0043	Dimer 4	0.1180	0.0067	Dimer 3	0.4410	0.0061	Dimer 5
11	0.0807	0.0045	Dimer 3	0.1266	0.0067	Dimer 4	0.1820	0.0139	Dimer 1
12	0.0811	0.0045	Dimer 4	0.0295	0.0070	Dimer 1	0.1858	0.0139	Dimer 2
13	0.0614	0.0047	Dimer 1	0.0296	0.0070	Dimer 2	0.0449	0.0141	Dimer 5
14	0.0616	0.0047	Dimer 2	0.0452	0.0074	Dimer 1	0.0467	0.0141	Dimer 5
15	0.0341	0.0050	Dimer 1	0.0463	0.0074	Dimer 2	0.2030	0.0172	Dimer 1
16	0.0342	0.0050	Dimer 2	0.0272	0.0074	Dimer 3	0.1925	0.0172	Dimer 2
17	0.0543	0.0052	Dimer 5	0.0240	0.0074	Dimer 4	0.1670	0.0178	Dimer 3
18	0.0091	0.0052	Dimer 5	0.0192	0.0075	Dimer 3	0.1634	0.0178	Dimer 4
19	0.0004	0.0052	Dimer 3	0.0243	0.0075	Dimer 4	0.1170	0.0183	Dimer 1
20	0.0004	0.0052	Dimer 4	0.0234	0.0076	Dimer 3	0.1140	0.0183	Dimer 2
21	0.0367	0.0468	Dimer 1	0.0288	0.0076	Dimer 4	0.0048	0.0183	Dimer 3
22	0.0368	0.0468	Dimer 2	0.0447	0.0076	Dimer 1	0.0094	0.0183	Dimer 4
23	0.0272	0.0473	Dimer 1	0.0444	0.0076	Dimer 2	0.0231	0.0185	Dimer 3
24	0.0273	0.0473	Dimer 2	0.0126	0.0076	Dimer 3	0.0159	0.0185	Dimer 4
25	0.0041	0.0475	Dimer 5	0.0120	0.0076	Dimer 4	0.0112	0.0185	Dimer 3
26	0.0001	0.0483	Dimer 3	0.0200	0.0077	Dimer 1	0.0179	0.0185	Dimer 4
27	0.0000	0.0483	Dimer 4	0.0195	0.0077	Dimer 2	0.0628	0.0186	Dimer 1
28	0.0355	0.0484	Dimer 5	0.0532	0.0082	Dimer 5	0.0627	0.0186	Dimer 2
29	0.0691	0.0486	Dimer 3	0.0117	0.0082	Dimer 5	0.0518	0.0190	Dimer 5
30	0.0692	0.0486	Dimer 4	0.0109	0.0082	Dimer 5	0.0518	0.0190	Dimer 5
31	0.0556	0.0653	Dimer 1	0.1900	0.0180	Dimer 5	1.4300	0.0210	Dimer 5
32	0.0558	0.0653	Dimer 2	0.2370	0.0193	Dimer 1	1.3100	0.0231	Dimer 1
33	0.0833	0.0653	Dimer 1	0.2440	0.0193	Dimer 2	1.3198	0.0231	Dimer 2
34	0.0835	0.0653	Dimer 2	0.0068	0.0207	Dimer 3	0.0161	0.0241	Dimer 3
35	0.0941	0.0662	Dimer 5	0.0031	0.0207	Dimer 4	0.0868	0.0241	Dimer 4
36	0.0111	0.0672	Dimer 5	0.1350	0.0209	Dimer 1	2.3300	0.0241	Dimer 3
37	0.0001	0.0673	Dimer 3	0.1271	0.0209	Dimer 2	2.2552	0.0241	Dimer 4
38	0.0001	0.0673	Dimer 4	0.4170	0.0210	Dimer 3	1.5100	0.0242	Dimer 1
39	0.0001	0.0676	Dimer 3	0.4471	0.0210	Dimer 4	1.4914	0.0242	Dimer 2
40	0.1509	0.0676	Dimer 4	0.1760	0.0214	Dimer 5	1.2300	0.0242	Dimer 5

Table S4. Summary of $|H_h|$ and $|H_e|$ of dimer 1–5 of $C(C_6H_5)_4$, $Si(C_6H_5)_4$, and $Ge(C_6H_5)_4$ crystals.

$C(C_6H_5)_4$

		Dimer 1	Dimer 2	Dimer 3	Dimer 4	Dimer 5
$\|H_h\|$	(eV)	4.56×10^{-3}	6.51×10^{-3}	2.22×10^{-3}	1.09×10^{-3}	1.87×10^{-3}
$\|H_e\|$	(eV)	1.58×10^{-2}	1.58×10^{-2}	1.25×10^{-2}	1.25×10^{-2}	3.19×10^{-2}
H_h^2	(eV^2)	2.08×10^{-5}	4.24×10^{-5}	4.93×10^{-6}	1.19×10^{-6}	3.50×10^{-6}
H_e^2	(eV^2)	2.49×10^{-4}	2.49×10^{-4}	1.56×10^{-4}	1.56×10^{-4}	1.02×10^{-3}
$H_h^2H_e^2$	(eV^4)	5.18×10^{-9}	1.06×10^{-8}	7.68×10^{-10}	1.85×10^{-10}	3.56×10^{-9}

$Si(C_6H_5)_4$

		Dimer 1	Dimer 2	Dimer 3	Dimer 4	Dimer 5
$\|H_h\|$	(eV)	3.73×10^{-3}	7.02×10^{-3}	9.40×10^{-4}	4.40×10^{-4}	8.17×10^{-3}
$\|H_e\|$	(eV)	8.45×10^{-3}	8.45×10^{-3}	3.01×10^{-3}	3.01×10^{-3}	5.03×10^{-2}
H_h^2	(eV^2)	1.39×10^{-5}	4.93×10^{-5}	8.84×10^{-7}	1.94×10^{-7}	6.67×10^{-5}
H_e^2	(eV^2)	7.14×10^{-5}	7.14×10^{-5}	9.06×10^{-6}	9.06×10^{-6}	2.53×10^{-3}
$H_h^2H_e^2$	(eV^4)	9.93×10^{-10}	3.52×10^{-9}	8.01×10^{-12}	1.75×10^{-12}	1.69×10^{-7}

$Ge(C_6H_5)_4$

		Dimer 1	Dimer 2	Dimer 3	Dimer 4	Dimer 5
$\|H_h\|$	(eV)	3.01×10^{-3}	1.45×10^{-2}	4.60×10^{-4}	1.91×10^{-3}	7.32×10^{-3}
$\|H_e\|$	(eV)	7.26×10^{-3}	7.26×10^{-3}	1.97×10^{-3}	1.97×10^{-3}	3.75×10^{-2}
H_h^2	(eV^2)	9.06×10^{-6}	2.10×10^{-4}	2.12×10^{-7}	3.65×10^{-6}	5.36×10^{-5}
H_e^2	(eV^2)	5.27×10^{-5}	5.27×10^{-5}	3.88×10^{-6}	3.88×10^{-6}	1.41×10^{-3}
$H_h^2H_e^2$	(eV^4)	4.78×10^{-10}	1.11×10^{-8}	8.21×10^{-13}	1.42×10^{-11}	7.53×10^{-8}

Table S5. Spin–orbit matrix elements ($|\langle \Psi_m^1|\overline{H_{SO}}|\Psi_n^3\rangle|^2$ (cm^{-2})) for dimer 2 in (a) $C(C_6H_5)_4$, (b) $Si(C_6H_5)_4$, and (c) $Ge(C_6H_5)_4$. Red values in (b) are $|\langle \Psi_m^1|\overline{H_{SO}}|\Psi_n^3\rangle|^2$>1.7 cm^{-2}. Red values in (c) are $|\langle \Psi_m^1|\overline{H_{SO}}|\Psi_n^3\rangle|^2$>5.1 cm^{-2}.

(a)

n \ m	1	2	3	4	5	6	7	8	9	10
1	0.031	0.027	0.018	0.275	0.051	0.381	0.000	0.028	0.020	0.012
2	0.001	0.066	0.011	0.301	0.014	0.090	0.006	0.067	0.091	0.005
3	0.477	0.136	0.001	0.052	0.079	0.128	0.074	0.123	0.071	0.005
4	0.151	0.452	0.000	0.057	0.012	0.453	0.001	0.003	0.061	0.057
5	0.447	0.193	0.023	0.116	0.009	0.003	0.000	0.078	0.021	0.007
6	0.082	0.385	0.003	0.322	0.010	0.001	0.000	0.023	0.083	0.018
7	0.087	0.844	0.020	0.444	0.003	0.017	0.003	0.037	0.132	0.022
8	0.906	0.102	0.094	0.611	0.011	0.085	0.000	0.058	0.020	0.045

(b)

n \ m	1	2	3	4	5	6	7	8	9	10
1	0.074	0.158	0.001	0.055	0.064	0.194	0.001	0.037	0.317	0.433
2	0.030	0.056	0.001	0.001	0.098	0.062	0.001	0.018	0.238	0.298
3	0.073	0.032	0.000	0.140	0.008	0.117	0.000	0.038	0.252	0.102
4	0.028	0.089	0.001	0.019	0.155	0.152	0.000	0.006	0.023	0.236
5	0.048	0.040	0.003	0.020	0.227	0.021	0.001	0.004	0.132	0.400
6	0.100	0.008	0.000	0.041	0.155	0.183	0.001	0.072	0.337	0.031
7	0.081	0.329	0.007	0.229	0.022	0.079	0.000	0.011	0.212	1.800
8	0.318	0.123	0.004	0.241	0.063	0.011	0.000	0.020	2.160	0.351

(c)

n \ m	1	2	3	4	5	6	7	8	9	10
1	0.464	0.038	0.000	0.205	0.013	0.030	0.030	0.076	5.370	1.860
2	0.014	0.271	0.016	0.091	0.000	0.009	0.012	0.769	2.460	2.650
3	0.363	0.052	0.039	0.117	0.037	0.224	0.006	0.074	0.867	0.879
4	0.133	0.398	0.001	0.041	0.088	0.172	0.001	0.042	0.160	1.330
5	0.184	0.204	0.001	0.006	0.192	0.008	0.000	0.032	0.443	0.627
6	0.014	0.032	0.004	0.425	0.123	0.060	0.001	0.098	0.504	0.124
7	0.670	0.311	0.044	0.509	0.090	0.508	0.000	0.015	5.880	6.630
8	0.180	0.943	0.039	0.620	0.392	0.083	0.000	0.005	6.530	7.510

Table S6. Spin–orbit matrix elements ($\left|\left\langle\Psi_m^1\middle|\overline{H_{SO}}\middle|\Psi_i^3\right\rangle\right|^2$ (cm^{-2})) for dimer 1–5 of $C(C_6H_5)_4$. The relationship between triplet energy and *i* is shown in Table S3.

i \ *m*	1	2	3	4	5	6	7	8	9	10	Number of dimer
1	0.106	0.463	0.044	0.263	0.498	0.639	0.045	0.304	1.180	0.194	Dimer 1
2	0.031	0.027	0.018	0.275	0.051	0.381	0.000	0.028	0.020	0.012	Dimer 2
3	0.001	0.066	0.011	0.301	0.014	0.090	0.007	0.065	0.091	0.004	Dimer 1
4	0.001	0.066	0.011	0.301	0.014	0.090	0.006	0.067	0.091	0.005	Dimer 2
5	0.002	0.196	0.000	0.542	0.042	0.002	0.046	0.372	0.034	0.015	Dimer 5
6	0.196	0.002	0.006	0.000	0.002	0.042	0.045	0.375	0.014	0.033	Dimer 5
7	0.066	0.001	0.012	0.006	0.005	0.603	0.006	0.002	0.139	0.004	Dimer 3
8	0.066	0.000	0.013	0.000	0.000	0.602	0.006	0.002	0.141	0.004	Dimer 4
9	0.001	0.020	0.000	0.645	0.612	0.005	0.000	0.081	0.001	0.149	Dimer 3
10	0.000	0.021	0.000	0.650	0.618	0.000	0.000	0.079	0.000	0.153	Dimer 4
11	0.625	0.000	0.003	0.000	0.000	0.617	0.021	0.001	0.083	0.007	Dimer 3
12	0.624	0.000	0.003	0.000	0.000	0.614	0.022	0.000	0.086	0.006	Dimer 4
13	0.477	0.136	0.001	0.052	0.079	0.127	0.074	0.121	0.070	0.006	Dimer 1
14	0.477	0.136	0.001	0.052	0.079	0.128	0.074	0.123	0.071	0.005	Dimer 2
15	0.151	0.452	0.000	0.057	0.013	0.452	0.001	0.003	0.062	0.056	Dimer 1
16	0.151	0.452	0.000	0.057	0.012	0.453	0.001	0.003	0.061	0.057	Dimer 2
17	0.000	0.467	0.000	0.202	0.000	0.000	0.035	0.254	0.042	0.095	Dimer 5
18	0.466	0.000	0.000	0.000	0.000	0.000	0.036	0.254	0.094	0.041	Dimer 5
19	0.000	0.672	0.000	0.023	0.623	0.000	0.000	0.140	0.005	0.080	Dimer 3
20	0.000	0.672	0.000	0.023	0.624	0.000	0.000	0.144	0.004	0.077	Dimer 4
21	0.447	0.193	0.023	0.117	0.009	0.003	0.000	0.079	0.019	0.008	Dimer 1
22	0.447	0.193	0.023	0.116	0.009	0.003	0.000	0.078	0.021	0.007	Dimer 2
23	0.082	0.385	0.003	0.322	0.010	0.001	0.000	0.023	0.081	0.018	Dimer 1
24	0.082	0.385	0.003	0.322	0.010	0.001	0.000	0.023	0.083	0.018	Dimer 2
25	0.476	0.491	0.000	0.007	0.004	0.004	0.000	0.000	0.063	0.068	Dimer 5
26	0.000	0.498	0.000	0.494	0.000	0.000	0.000	0.116	0.000	0.125	Dimer 3
27	0.000	0.498	0.000	0.495	0.000	0.000	0.000	0.116	0.000	0.125	Dimer 4
28	0.041	0.029	0.053	0.448	0.003	0.003	0.000	0.001	0.038	0.035	Dimer 5
29	0.514	0.000	0.090	0.000	0.000	0.000	0.000	0.001	0.118	0.000	Dimer 3
30	0.514	0.000	0.089	0.000	0.000	0.000	0.000	0.000	0.118	0.000	Dimer 4
31	0.086	0.844	0.020	0.444	0.003	0.017	0.003	0.037	0.128	0.023	Dimer 1
32	0.087	0.844	0.020	0.444	0.003	0.017	0.003	0.037	0.132	0.022	Dimer 2
33	0.906	0.102	0.094	0.611	0.011	0.086	0.000	0.056	0.019	0.044	Dimer 1
34	0.906	0.102	0.094	0.611	0.011	0.085	0.000	0.058	0.020	0.045	Dimer 2
35	0.023	0.021	0.001	1.000	0.000	0.000	0.012	0.000	0.075	0.072	Dimer 5
36	0.023	0.021	0.001	1.000	0.000	0.000	0.012	0.000	0.075	0.072	Dimer 5
37	0.000	1.050	0.000	1.010	0.098	0.000	0.000	0.130	0.000	0.155	Dimer 3
38	0.000	1.050	0.000	1.010	0.098	0.000	0.000	0.132	0.000	0.151	Dimer 4
39	1.040	0.000	0.005	0.000	0.000	0.094	0.001	0.001	0.147	0.000	Dimer 3
40	1.040	0.000	0.005	0.000	0.000	0.094	0.000	0.001	0.152	0.000	Dimer 4

Table S7. Spin–orbit matrix elements ($\left|\left\langle \Psi_m^1 \middle| \overline{H_{SO}} \middle| \Psi_i^3 \right\rangle\right|^2$ (cm^{-2})) for dimer 1–5 of $Si(C_6H_5)_4$. The relationship between triplet energy and *i* is shown in Table S3. Red values are $|\langle \Psi_m^1 | \overline{H_{SO}} | \Psi_n^3 \rangle|^2 > 1.7\ cm^{-2}$.

i \ m	1	2	3	4	5	6	7	8	9	10	Number of dimer
1	0.074	0.158	0.001	0.055	0.064	0.194	0.001	0.038	0.347	0.413	Dimer 5
2	0.181	0.180	0.000	0.047	0.047	0.006	0.010	0.523	0.059	0.057	Dimer 1
3	0.074	0.158	0.001	0.055	0.064	0.194	0.001	0.037	0.317	0.433	Dimer 2
4	0.031	0.056	0.001	0.001	0.098	0.062	0.001	0.018	0.226	0.314	Dimer 5
5	0.073	0.032	0.000	0.139	0.008	0.117	0.000	0.038	0.257	0.092	Dimer 5
6	0.023	0.022	0.009	0.004	0.068	0.122	0.000	0.000	0.365	0.012	Dimer 1
7	0.030	0.056	0.001	0.001	0.098	0.062	0.001	0.018	0.238	0.298	Dimer 2
8	0.095	0.033	0.000	0.122	0.000	0.131	0.000	0.002	0.376	0.079	Dimer 3
9	0.095	0.033	0.000	0.122	0.000	0.131	0.000	0.003	0.380	0.063	Dimer 4
10	0.008	0.162	0.007	0.000	0.036	0.000	0.000	0.014	0.038	0.696	Dimer 3
11	0.008	0.162	0.007	0.000	0.036	0.000	0.000	0.015	0.032	0.700	Dimer 4
12	0.022	0.023	0.009	0.068	0.004	0.121	0.000	0.046	0.012	0.366	Dimer 1
13	0.073	0.032	0.000	0.140	0.008	0.117	0.000	0.038	0.252	0.102	Dimer 2
14	0.058	0.058	0.000	0.120	0.120	0.000	0.040	0.364	0.304	0.302	Dimer 1
15	0.028	0.089	0.001	0.019	0.155	0.152	0.000	0.006	0.023	0.236	Dimer 2
16	0.082	0.070	0.000	0.058	0.000	0.253	0.001	0.001	0.356	0.166	Dimer 3
17	0.082	0.071	0.000	0.058	0.000	0.253	0.001	0.000	0.352	0.134	Dimer 4
18	0.030	0.001	0.000	0.064	0.389	0.008	0.000	0.089	0.029	0.029	Dimer 3
19	0.032	0.002	0.000	0.063	0.391	0.005	0.000	0.089	0.034	0.025	Dimer 4
20	0.001	0.007	0.006	0.002	0.008	0.390	0.000	0.001	0.009	0.181	Dimer 3
21	0.001	0.007	0.006	0.001	0.005	0.393	0.000	0.001	0.007	0.235	Dimer 4
22	0.061	0.001	0.000	0.058	0.000	0.169	0.000	0.222	0.003	0.046	Dimer 1
23	0.048	0.040	0.003	0.020	0.227	0.021	0.001	0.004	0.132	0.400	Dimer 2
24	0.072	0.023	0.001	0.028	0.354	0.000	0.000	0.068	0.369	0.074	Dimer 3
25	0.070	0.023	0.001	0.030	0.355	0.000	0.000	0.064	0.367	0.076	Dimer 4
26	0.001	0.061	0.000	0.000	0.058	0.170	0.001	0.022	0.046	0.003	Dimer 1
27	0.100	0.008	0.000	0.041	0.155	0.183	0.001	0.072	0.337	0.031	Dimer 2
28	0.028	0.089	0.001	0.019	0.156	0.151	0.001	0.005	0.029	0.235	Dimer 5
29	0.048	0.040	0.004	0.020	0.227	0.020	0.000	0.003	0.120	0.412	Dimer 5
30	0.099	0.008	0.000	0.041	0.153	0.184	0.001	0.071	0.335	0.026	Dimer 5
31	0.081	0.329	0.007	0.229	0.022	0.079	0.000	0.010	0.170	1.810	Dimer 5
32	0.094	0.094	0.000	0.362	0.363	0.021	0.007	0.512	1.400	1.400	Dimer 1
33	0.081	0.329	0.007	0.229	0.022	0.079	0.000	0.011	0.212	1.800	Dimer 2
34	0.525	0.000	0.000	0.419	0.029	0.004	0.001	0.000	2.540	0.022	Dimer 3
35	0.525	0.000	0.000	0.418	0.029	0.004	0.001	0.001	2.550	0.012	Dimer 4
36	0.340	0.339	0.000	0.221	0.222	0.000	0.030	0.741	1.190	1.180	Dimer 1
37	0.318	0.123	0.004	0.241	0.063	0.011	0.000	0.020	2.160	0.351	Dimer 2
38	0.000	0.510	0.003	0.000	0.001	0.025	0.000	0.007	0.029	2.550	Dimer 3
39	0.000	0.510	0.003	0.000	0.001	0.025	0.000	0.011	0.009	2.570	Dimer 4
40	0.081	0.329	0.007	0.229	0.022	0.079	0.000	0.010	0.170	1.810	Dimer 5

Table S8. Spin–orbit matrix elements ($|\langle\Psi_m^1|\overline{H_{SO}}|\Psi_i^3\rangle|^2$ (cm^{-2})) for dimer 1–5 of $Ge(C_6H_5)_4$. The relationship between triplet energy and i is shown in Table S3. Red values are $|\langle\Psi_m^1|\overline{H_{SO}}|\Psi_n^3\rangle|^2$>5.1 cm^{-2}.

i \ m	1	2	3	4	5	6	7	8	9	10	Number of dimer
1	0.261	0.261	0.000	0.226	0.226	0.079	0.168	4.710	0.645	0.646	Dimer 5
2	0.464	0.037	0.000	0.205	0.013	0.030	0.030	0.077	5.390	1.800	Dimer 1
3	0.464	0.038	0.000	0.205	0.013	0.030	0.030	0.076	5.370	1.860	Dimer 2
4	0.014	0.271	0.016	0.091	0.000	0.009	0.012	0.768	2.450	2.650	Dimer 1
5	0.014	0.271	0.016	0.091	0.000	0.009	0.012	0.769	2.460	2.650	Dimer 2
6	0.288	0.001	0.000	0.254	0.001	0.001	0.009	0.002	5.850	0.017	Dimer 3
7	0.353	0.001	0.000	0.223	0.001	0.000	0.009	0.003	8.420	0.122	Dimer 4
8	0.000	0.291	0.006	0.001	0.000	0.000	0.000	0.946	0.025	5.970	Dimer 3
9	0.104	0.233	0.004	0.049	0.000	0.000	0.000	0.946	2.890	5.850	Dimer 4
10	0.167	0.167	0.027	0.155	0.156	0.000	0.097	2.500	2.340	2.350	Dimer 5
11	0.363	0.052	0.039	0.117	0.037	0.224	0.005	0.074	0.903	0.829	Dimer 1
12	0.363	0.052	0.039	0.117	0.037	0.224	0.006	0.074	0.867	0.879	Dimer 2
13	0.474	0.000	0.087	0.008	0.090	0.078	0.006	0.015	0.502	0.028	Dimer 5
14	0.000	0.475	0.086	0.090	0.008	0.080	0.000	0.271	0.029	0.502	Dimer 5
15	0.133	0.398	0.001	0.041	0.088	0.172	0.001	0.042	0.170	1.370	Dimer 1
16	0.133	0.398	0.001	0.041	0.088	0.172	0.001	0.042	0.160	1.330	Dimer 2
17	0.017	0.057	0.003	0.001	0.002	0.226	0.000	0.081	0.115	1.050	Dimer 3
18	0.114	0.057	0.003	0.049	0.002	0.138	0.000	0.043	2.860	1.050	Dimer 4
19	0.184	0.204	0.001	0.006	0.192	0.008	0.000	0.032	0.429	0.656	Dimer 1
20	0.184	0.204	0.001	0.006	0.192	0.008	0.000	0.032	0.443	0.627	Dimer 2
21	0.034	0.003	0.000	0.529	0.228	0.000	0.001	0.002	0.959	0.028	Dimer 3
22	0.139	0.002	0.000	0.577	0.146	0.000	0.001	0.003	3.730	0.046	Dimer 4
23	0.025	0.487	0.003	0.003	0.011	0.244	0.000	0.104	0.132	0.007	Dimer 3
24	0.234	0.389	0.001	0.060	0.045	0.175	0.000	0.071	2.890	0.022	Dimer 4
25	0.523	0.023	0.000	0.053	0.233	0.012	0.001	0.006	0.050	0.113	Dimer 3
26	0.523	0.121	0.000	0.091	0.161	0.048	0.001	0.023	2.850	0.115	Dimer 4
27	0.014	0.032	0.004	0.425	0.123	0.060	0.001	0.098	0.507	0.130	Dimer 1
28	0.014	0.032	0.004	0.425	0.123	0.060	0.001	0.098	0.504	0.124	Dimer 2
29	0.000	0.104	0.000	0.459	0.016	0.115	0.000	0.527	0.009	1.560	Dimer 5
30	0.104	0.000	0.000	0.016	0.460	0.116	0.004	0.000	0.044	0.132	Dimer 5
31	0.353	0.353	0.000	0.611	0.611	0.439	0.040	3.930	8.940	8.950	Dimer 5
32	0.670	0.311	0.044	0.509	0.090	0.508	0.000	0.015	6.040	6.440	Dimer 1
33	0.670	0.311	0.044	0.509	0.090	0.508	0.000	0.015	5.880	6.630	Dimer 2
34	1.250	0.001	0.000	0.962	0.461	0.018	0.000	0.008	15.200	0.043	Dimer 3
35	0.800	0.002	0.000	0.941	0.461	0.017	0.000	0.016	10.300	0.157	Dimer 4
36	0.001	1.210	0.109	0.000	0.007	0.494	0.000	0.001	0.062	15.400	Dimer 3
37	0.105	0.716	0.091	0.048	0.008	0.494	0.001	0.001	2.930	7.780	Dimer 4
38	0.180	0.943	0.039	0.620	0.392	0.083	0.000	0.005	6.380	7.740	Dimer 1
39	0.180	0.943	0.039	0.620	0.392	0.083	0.000	0.005	6.530	7.510	Dimer 2
40	0.353	0.353	0.000	0.611	0.611	0.439	0.040	3.930	8.940	8.950	Dimer 5

Table S9. Relationships between $\mu_{S_m \rightarrow S_0}{}^2$ (D^2) and m of dimer 1–5 in $C(C_6H_5)_4$, $Si(C_6H_5)_4$, and $Ge(C_6H_5)_4$.

$C(C_6H_5)_4$

Type \ *m*	1	2	3	4	5	6	7	8	9	10
Dimer 1	0.423	0.423	1.370	0.443	0.145	0.000	0.032	0.032	0.045	4.060
Dimer 2	0.831	0.257	0.222	0.518	0.439	0.389	2.070	0.389	0.585	0.044
Dimer 3	0.927	0.000	0.973	0.000	0.005	2.450	1.020	0.002	0.820	0.002
Dimer 4	0.927	0.000	0.973	0.000	0.007	2.460	1.000	0.001	0.848	0.002
Dimer 5	0.492	0.488	0.304	0.000	1.660	1.660	0.001	0.035	0.741	0.746

$Si(C_6H_5)_4$

Type \ *m*	1	2	3	4	5	6	7	8	9	10
Dimer 1	0.179	1.070	0.412	0.651	0.065	0.540	0.005	0.003	0.853	2.770
Dimer 2	0.179	1.070	0.412	0.651	0.065	0.549	0.005	0.005	0.741	2.870
Dimer 3	0.000	0.962	1.080	0.003	0.005	0.522	0.000	0.009	0.035	3.710
Dimer 4	0.000	0.962	1.080	0.003	0.005	0.522	0.001	0.011	0.012	3.980
Dimer 5	0.216	0.219	0.000	0.509	0.509	0.288	0.505	0.505	1.350	1.350

$Ge(C_6H_5)_4$

Type \ *m*	1	2	3	4	5	6	7	8	9	10
Dimer 1	0.092	0.480	0.230	0.435	0.003	0.423	0.002	0.003	2.070	3.510
Dimer 2	0.092	0.480	0.230	0.435	0.003	0.423	0.002	0.003	2.160	3.420
Dimer 3	0.000	0.505	0.531	0.001	0.001	0.371	0.009	0.001	0.017	4.110
Dimer 4	0.000	0.505	0.531	0.001	0.000	0.371	0.012	0.000	0.082	4.040
Dimer 5	0.123	0.123	0.000	0.245	0.245	0.285	0.700	0.705	1.880	1.880

Table S10. Summary of S_m-S_0 energies and $\Delta E_{T1\text{-}Sm}$ of dimer 2 of $C(C_6H_5)_4$, $Si(C_6H_5)_4$, and $Ge(C_6H_5)_4$.

$C(C_6H_5)_4$

m	1	2	3	4	5	6	7	8	9	10
S_m-S_0 energy (eV)	5.031	5.034	5.050	5.050	5.230	5.270	5.270	5.300	5.320	5.330
T_1-S_0 energy (eV)	3.960									
$\Delta E_{T1\text{-}Sm}$ (eV)	1.071	1.075	1.086	1.089	1.269	1.307	1.314	1.342	1.360	1.372

$Si(C_6H_5)_4$

m	1	2	3	4	5	6	7	8	9	10
S_m-S_0 energy (eV)	5.266	5.278	5.280	5.290	5.300	5.310	5.370	5.380	5.420	5.430
T_1-S_0 energy (eV)	4.035									
$\Delta E_{T1\text{-}Sm}$ (eV)	1.230	1.243	1.247	1.251	1.268	1.271	1.336	1.343	1.389	1.393

$Ge(C_6H_5)_4$

m	1	2	3	4	5	6	7	8	9	10
S_m-S_0 energy (eV)	5.327	5.338	5.340	5.340	5.370	5.370	5.400	5.410	5.510	5.520
T_1-S_0 energy (eV)	4.047									
$\Delta E_{T1\text{-}Sm}$ (eV)	1.281	1.292	1.292	1.297	1.322	1.325	1.357	1.363	1.465	1.477

5. Figure S1–S11

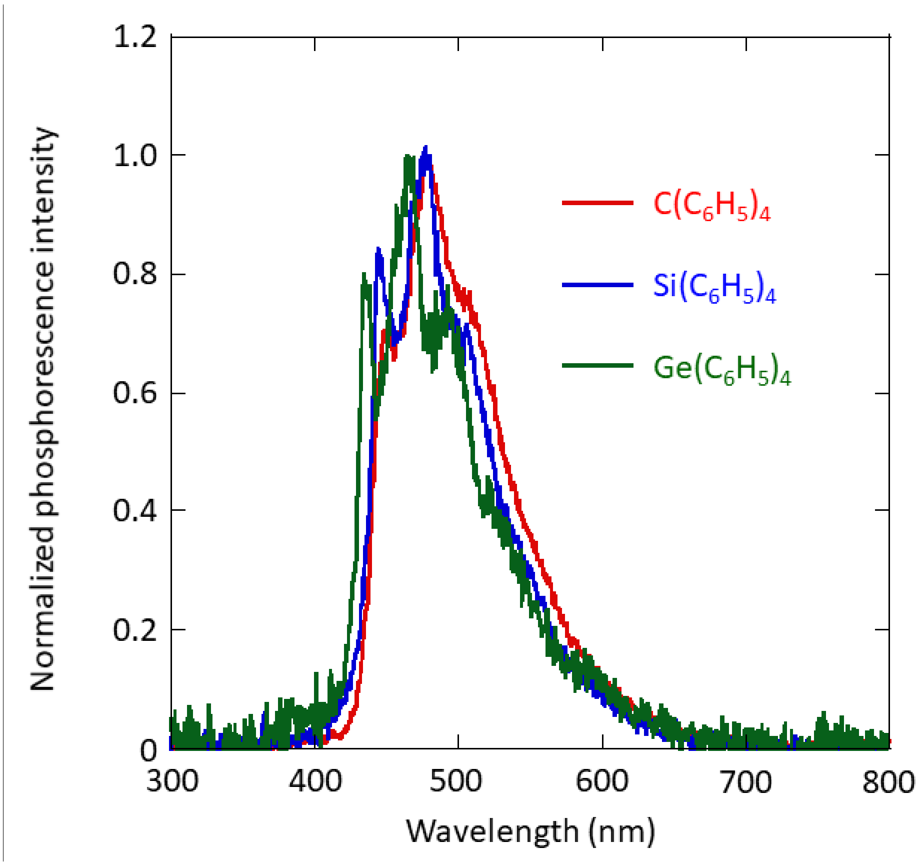

Figure S1. Phosphorescence spectra of $C(C_6H_5)_4$, $Si(C_6H_5)_4$, and $Ge(C_6H_5)_4$ in 2-methyl-tetrahydrofuran at 77 K. Spectra were measured soon after ceasing excitation at 280 nm.

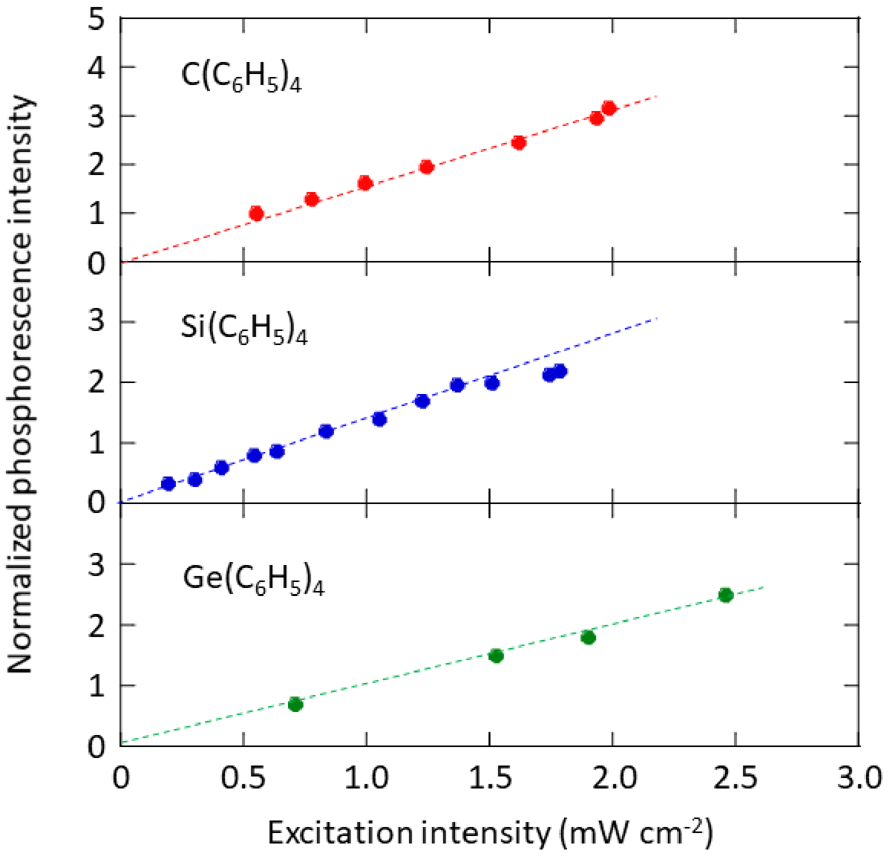

Figure S2. Dependence of RTP intensity change of $C(C_6H_5)_4$, $Si(C_6H_5)_4$, and $Ge(C_6H_5)_4$ crystals on excitation intensity at 280 nm.

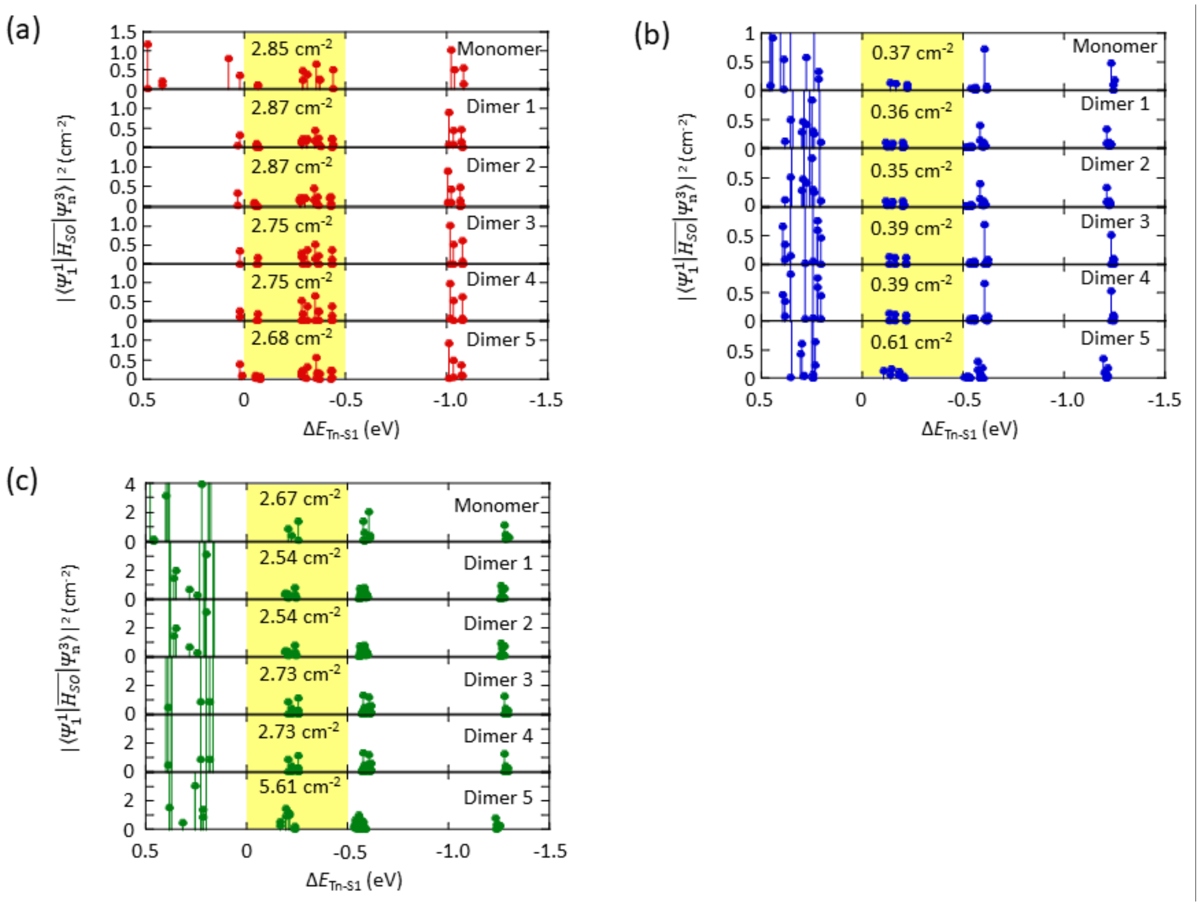

Figure S3. Relationships between $|\langle\Psi_1^1|\overline{H_{SO}}|\Psi_n^3\rangle|^2$ and $\Delta E_{\text{Tn-S1}}$ of monomers and dimer 1–5 of (a) $C(C_6H_5)_4$, (b) $Si(C_6H_5)_4$, and (c) $Ge(C_6H_5)_4$.

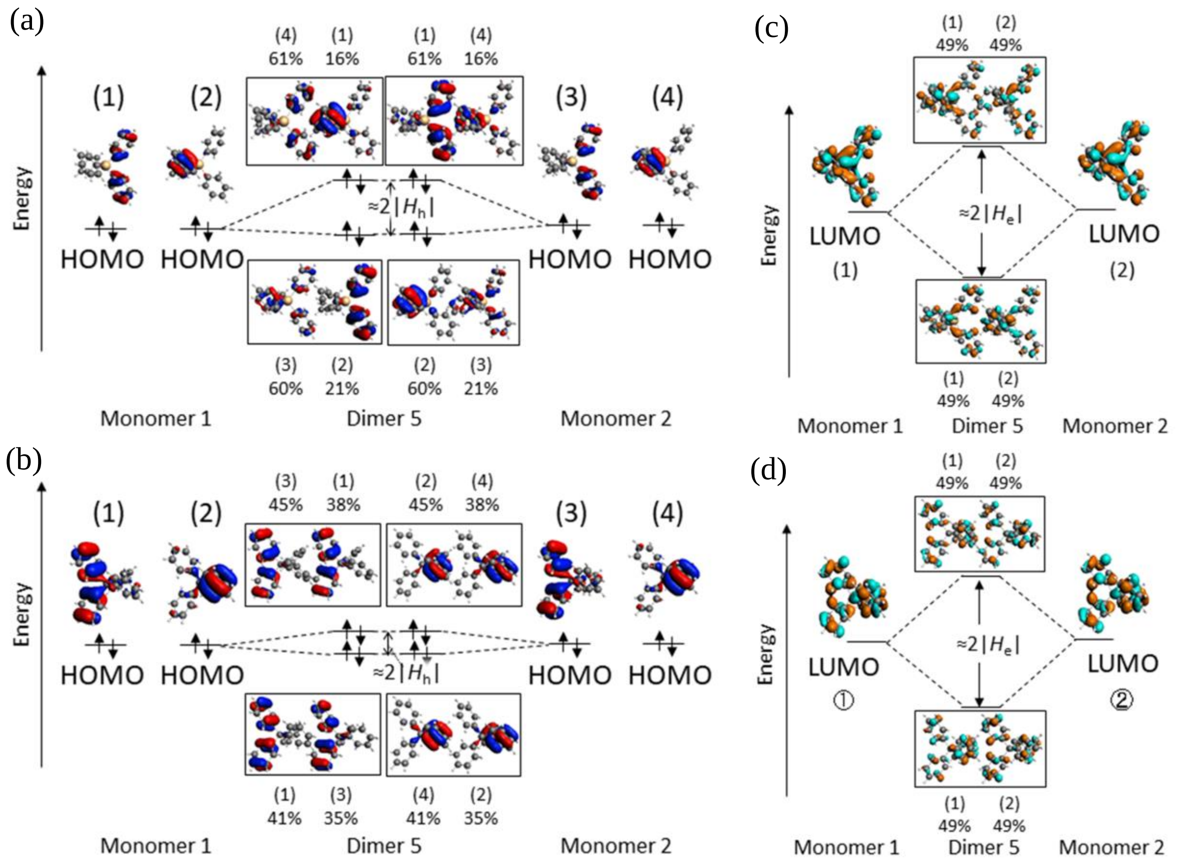

Figure S4. Structures of MOs causing small |H_h| and large |H_e| of Si(C_6H_5)$_4$ and C(C_6H_5)$_4$ crystals. (a) Electronic structures causing small |H_h| of dimer 5 of Si(C_6H_5)$_4$. (b) Electronic structures causing small |H_h| of dimer 5 of C(C_6H_5)$_4$. (c) Electronic structures causing large |H_e| of dimer 5 of Si(C_6H_5)$_4$. (d) Electronic structures causing large |H_e| of dimer 5 of C(C_6H_5)$_4$.

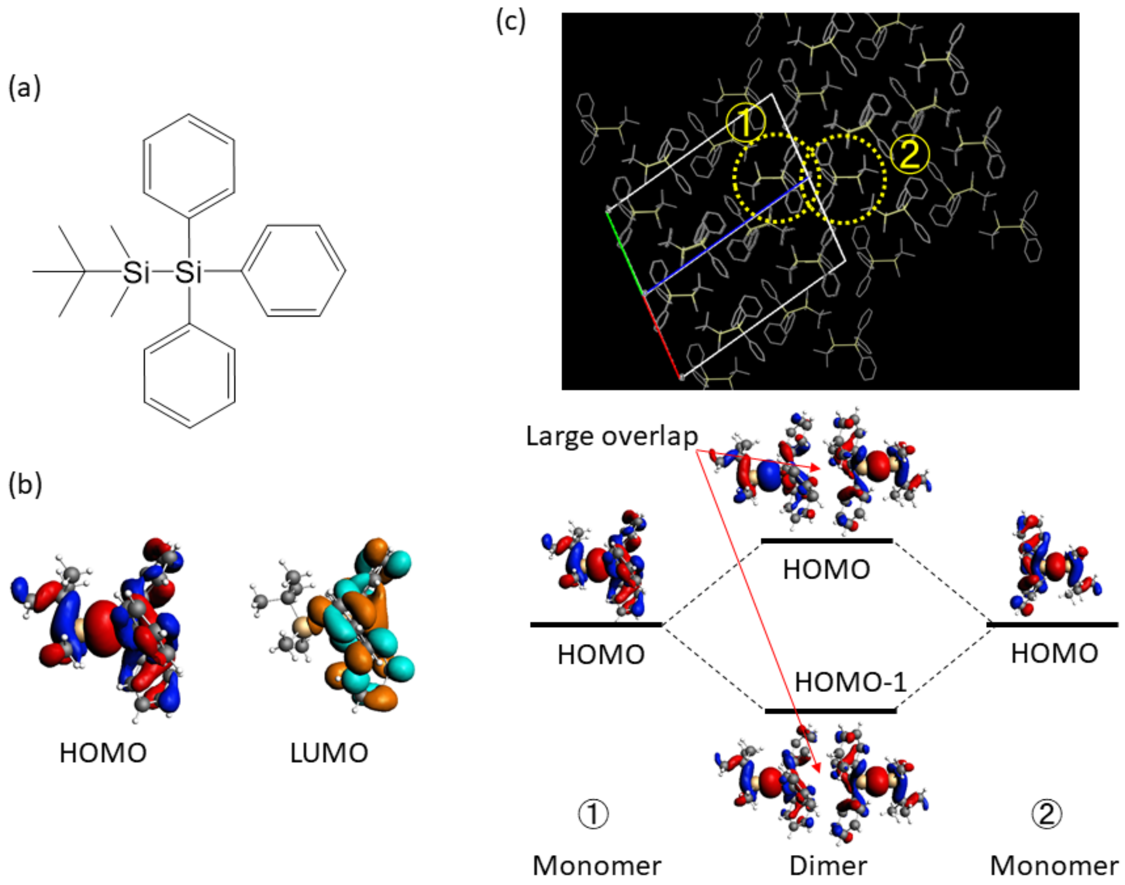

Figure S5. Molecular, electronic, and crystalline structures of (*tert*-butyldimethylsilyl)triphenylsilane. (a) Chemical structure. (b) HOMO and LUMO of a monomer. (c) Crystalline structure and molecular orbitals related to the hole transfer integral of a dimer in the crystal.

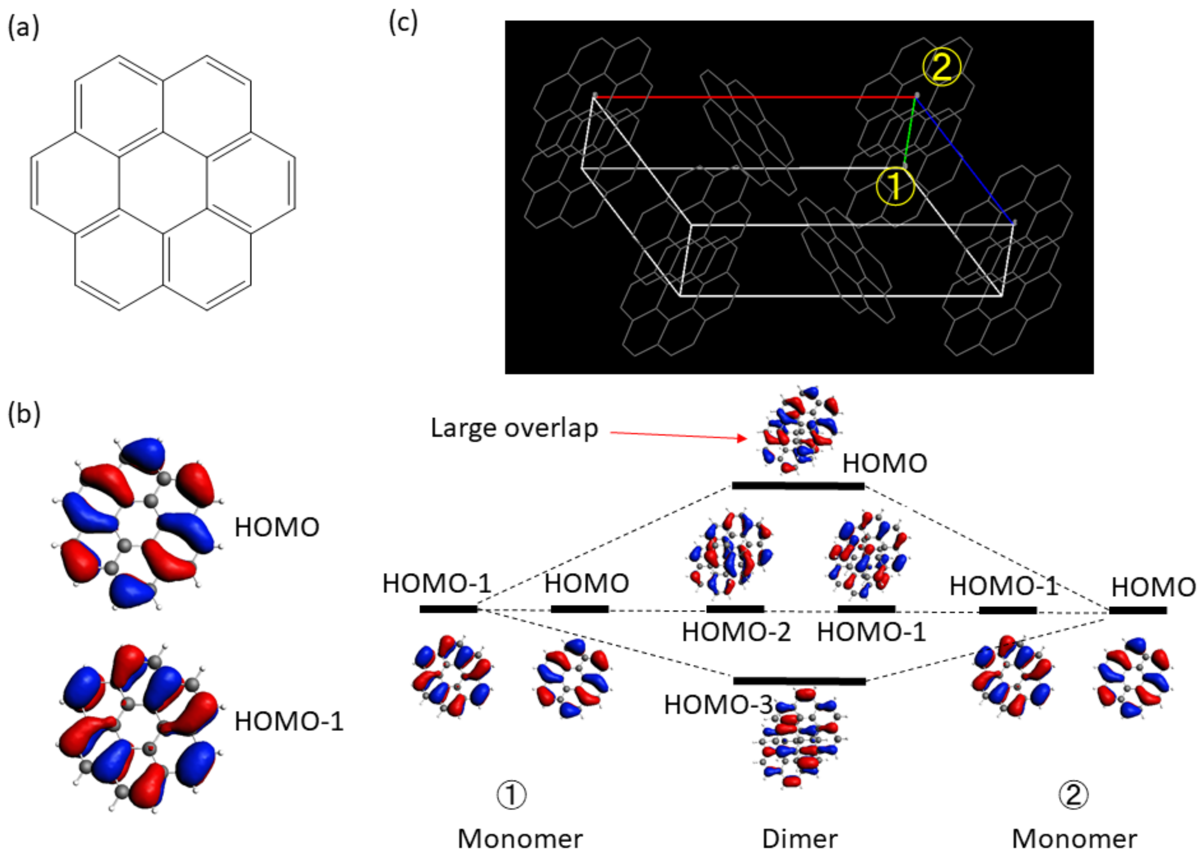

Figure S6. Molecular, electronic, and crystalline structures of coronene. (a) Chemical structure. (b) HOMO and LUMO of a monomer. (c) Crystalline structure and molecular orbitals related to hole transfer integral of a dimer in the crystal.

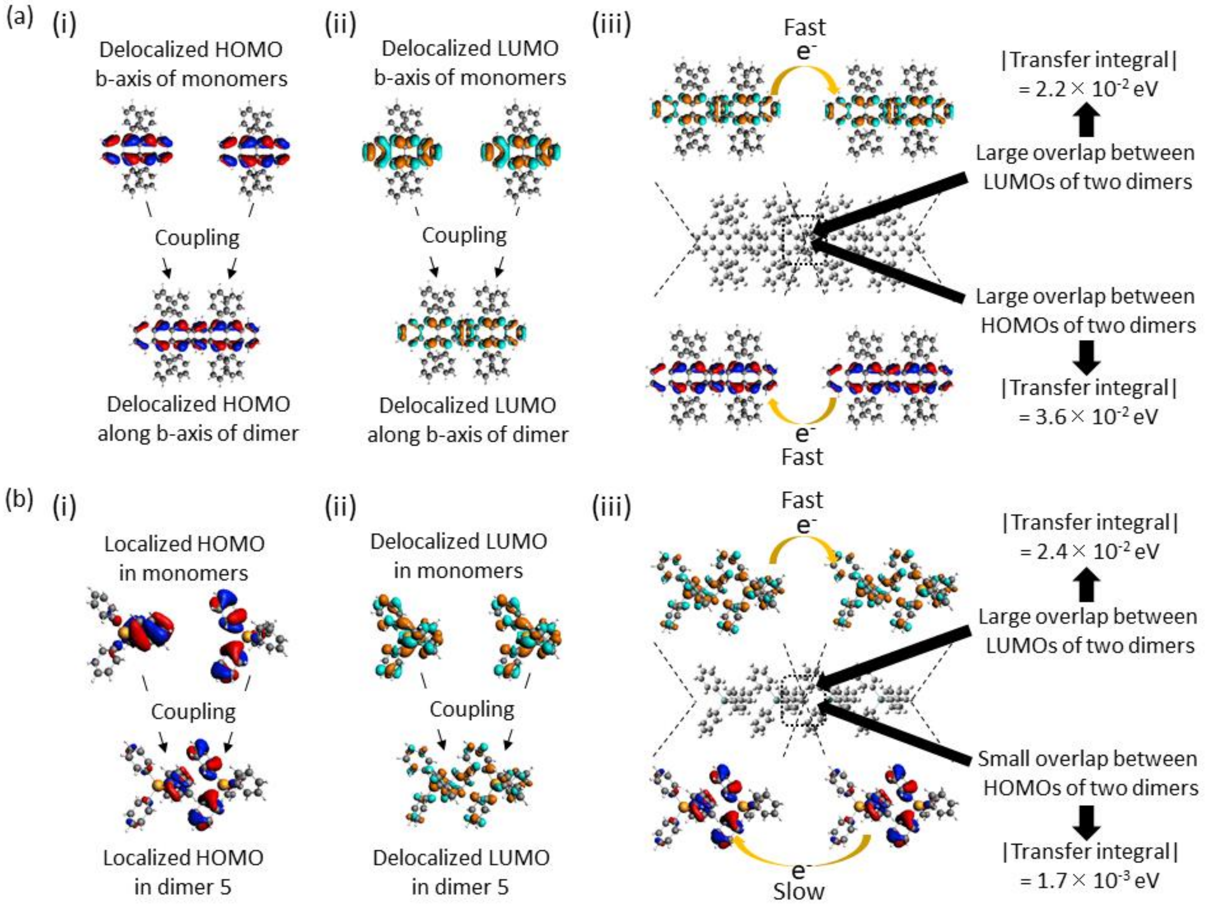

Figure S7. Dependence of the HOMOs and LUMOs of rubrene and $Ge(C_6H_5)_4$ on molecular stacking. (a) (i) Change of HOMO caused by dimerization of two rubrene monomers along the *b*-axis of a rubrene crystal. (ii) Change of LUMO caused by dimerization of two rubrene monomers along the *b*-axis of a rubrene crystal. (iii) Overlap of the HOMOs and LUMOs of each rubrene dimer in a tetramer along the *b*-axis of a rubrene crystal and hole and electron transfer integrals between the two dimers in the tetramer. (b) (i) Change of HOMO caused by dimerization of two $Ge(C_6H_5)_4$ monomers along the *c*-axis of a $Ge(C_6H_5)_4$ crystal. (ii) Change of LUMO caused by dimerization of two $Ge(C_6H_5)_4$ monomers along the *c*-axis of a $Ge(C_6H_5)_4$ crystal. (iii) Overlap of HOMOs and LUMOs of each $Ge(C_6H_5)_4$ dimer in a tetramer along the *c*-axis of a $Ge(C_6H_5)_4$ crystal and hole and electron transfer integrals between the two dimers in the tetramer.

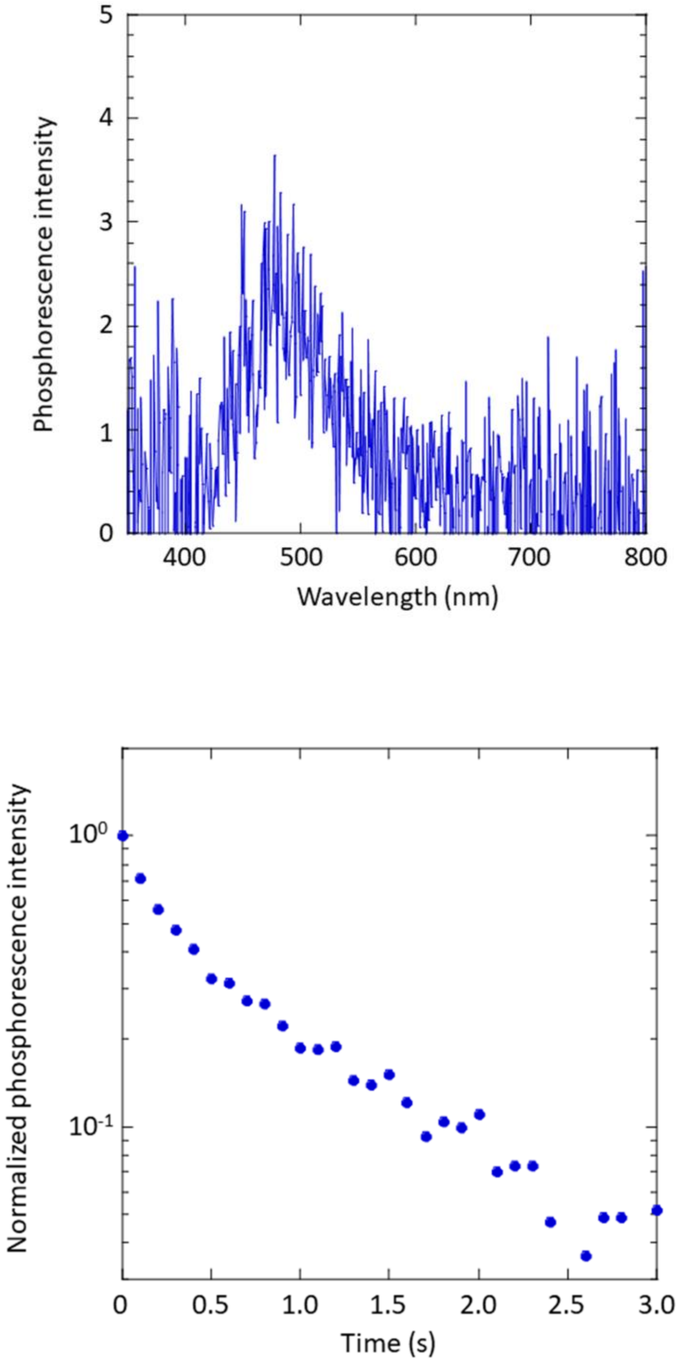

Figure S8. (a) RTP spectrum and (b) decay characteristics of a 1 wt% $Si(C_6H_5)_4$-doped Zeonex film under vacuum conditions. Excitation wavelength is 280 nm.

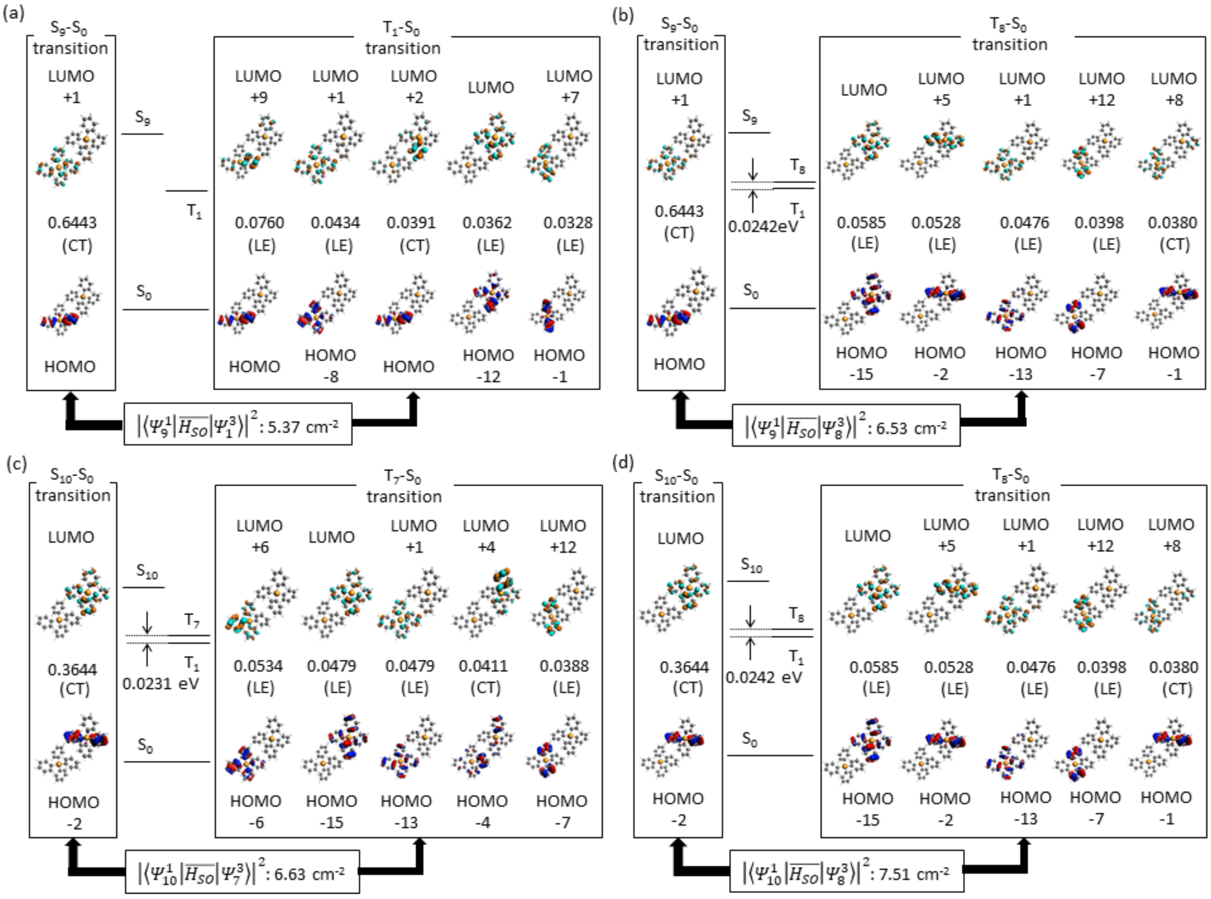

Figure S9. MOs related to the S_m–S_0 and T_n–S_0 transitions contributing to the large $|\langle\Psi_m^1|\overline{H_{SO}}|\Psi_n^3\rangle|^2$ of dimer 2 of $Ge(C_6H_5)_4$. (a) MOs related to the S_9–S_0 and T_1–S_0 transitions and value of $|\langle\Psi_9^1|\overline{H_{SO}}|\Psi_1^3\rangle|^2$. (b) MOs related to the S_9–S_0 and T_8–S_0 transitions and value of $|\langle\Psi_9^1|\overline{H_{SO}}|\Psi_8^3\rangle|^2$. (c) MOs related to the S_{10}–S_0 and T_7–S_0 transitions and value of $|\langle\Psi_{10}^1|\overline{H_{SO}}|\Psi_7^3\rangle|^2$. (d) MOs related to the S_{10}–S_0 and T_8–S_0 transitions and value of $|\langle\Psi_{10}^1|\overline{H_{SO}}|\Psi_8^3\rangle|^2$.

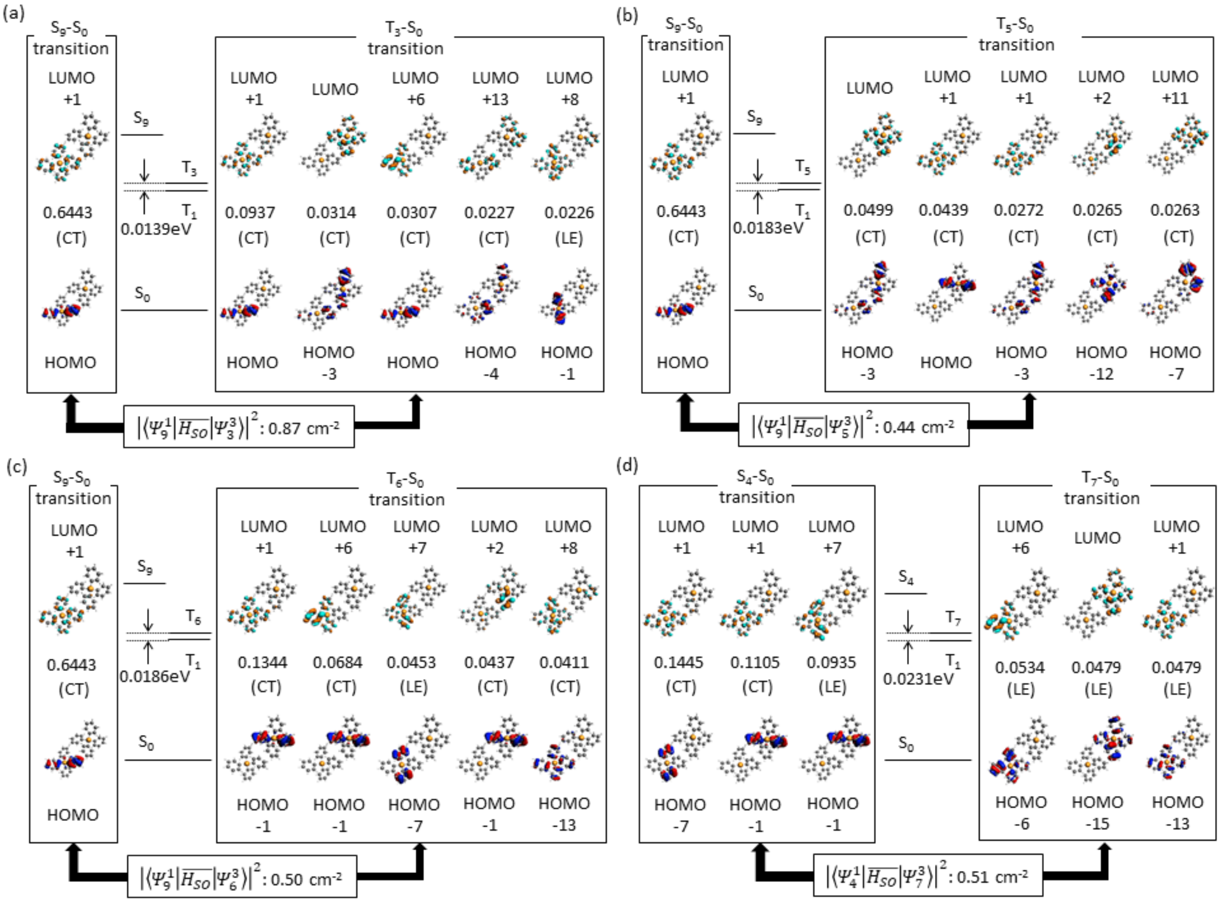

Figure S10. MOs related to the S_m–S_0 and T_n–S_0 transitions contributing to small $|\langle \Psi_m^1 | \overline{H_{SO}} | \Psi_n^3 \rangle|^2$. (a) MOs related to the S_9–S_0 and T_3–S_0 transitions and value of $|\langle \Psi_9^1 | \overline{H_{SO}} | \Psi_3^3 \rangle|^2$ for dimer 2 of $Ge(C_6H_5)_4$. (b) MOs related to the S_9–S_0 and T_5–S_0 transitions and value of $|\langle \Psi_9^1 | \overline{H_{SO}} | \Psi_5^3 \rangle|^2$ for dimer 2 of $Ge(C_6H_5)_4$. (c) MOs related to the S_9–S_0 and T_6–S_0 transitions and value of $|\langle \Psi_9^1 | \overline{H_{SO}} | \Psi_6^3 \rangle|^2$ for dimer 2 of $Ge(C_6H_5)_4$. (d) MOs related to the S_4–S_0 and T_7–S_0 transitions and value of $|\langle \Psi_4^1 | \overline{H_{SO}} | \Psi_7^3 \rangle|^2$ for dimer 2 of $Si(C_6H_5)_4$.

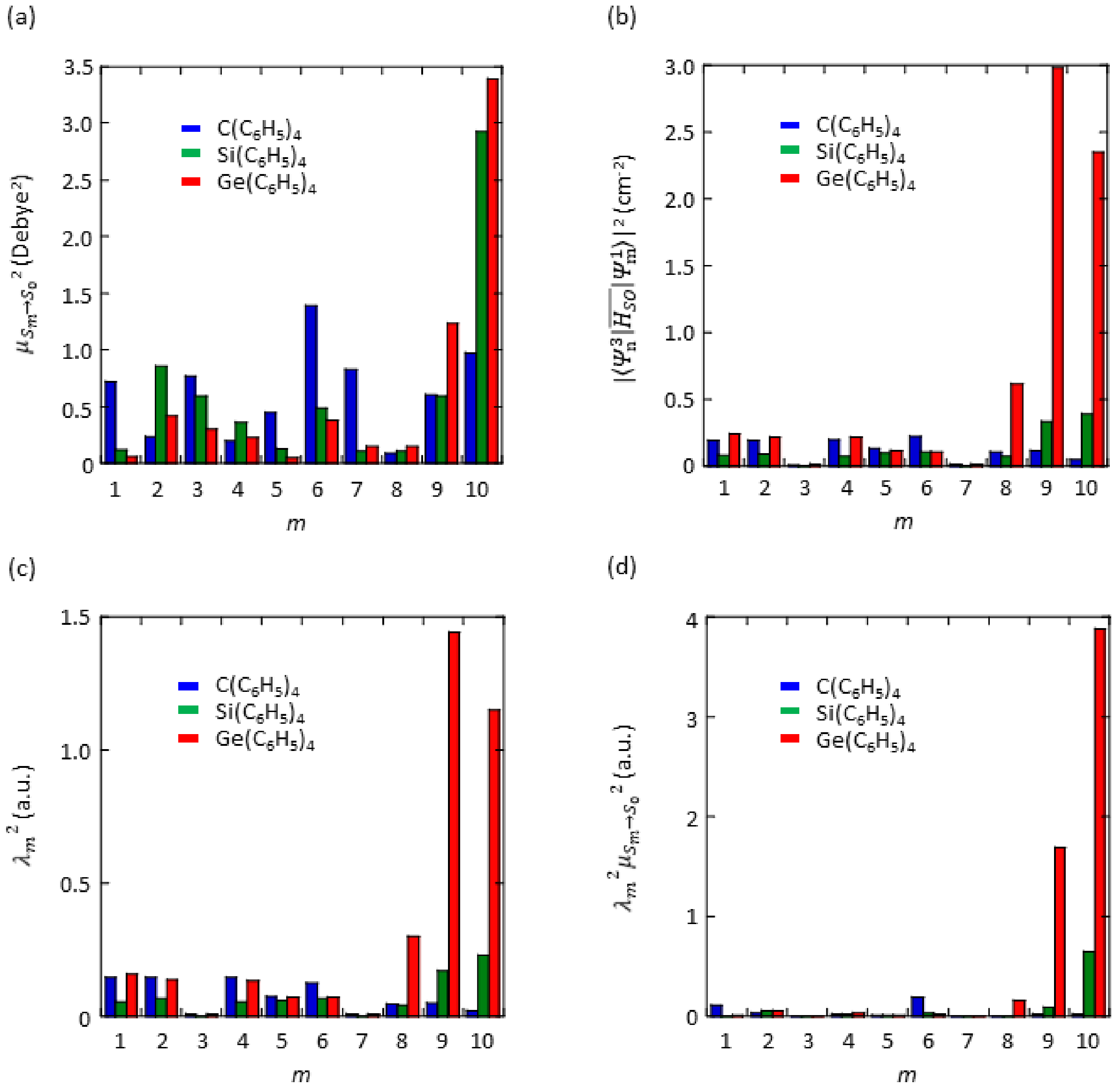

Figure S11. Relationships between photophysical parameters related to k_p and m for $C(C_6H_5)_4$, $Si(C_6H_5)_4$, and $Ge(C_6H_5)_4$ crystals. (a) Relationship between m and ${\mu_{S_m \to S_0}}^2$ averaged for i=1–40 of dimer 1–5. (b) Relationship between m and $\left|\langle \Psi_n^1 | \overline{H_{SO}} | \Psi_i^3 \rangle\right|^2$ averaged for i=1–40 of dimer 1–5. (c) Relationship between m and ${\lambda_m}^2$ averaged for i=1–40 of dimer 1–5. (d) Relationship between m and ${\mu_{S_m \to S_0}}^2 {\lambda_m}^2$ averaged for i=1–40 of dimer 1–5.

6. Supporting reference

(S1) K. Schmidt, S. Brovelli, V. Coropceanu, D. Baljonne, J. Cornil, C. Bazzini, T. Caronna, R. Tubino, F. Meinardi, Z. Shuai, J-L. Bredas, *J. Phys. Chem. A* **2007**, *111*, 10490.
